# Isolation and characterization of *Streptomyces* bacteriophages and *Streptomyces* strains encoding biosynthetic arsenals

**DOI:** 10.1371/journal.pone.0262354

**Published:** 2022-01-21

**Authors:** Elizabeth T. Montaño, Jason F. Nideffer, Lauren Brumage, Marcella Erb, Julia Busch, Lynley Fernandez, Alan I. Derman, John Paul Davis, Elena Estrada, Sharon Fu, Danielle Le, Aishwarya Vuppala, Cassidy Tran, Elaine Luterstein, Shivani Lakkaraju, Sriya Panchagnula, Caroline Ren, Jennifer Doan, Sharon Tran, Jamielyn Soriano, Yuya Fujita, Pranathi Gutala, Quinn Fujii, Minda Lee, Anthony Bui, Carleen Villarreal, Samuel R. Shing, Sean Kim, Danielle Freeman, Vipula Racha, Alicia Ho, Prianka Kumar, Kian Falah, Thomas Dawson, Eray Enustun, Amy Prichard, Ana Gomez, Kanika Khanna, Shelly A. Wanamaker, Kit Pogliano, Joe Pogliano

**Affiliations:** 1 Division of Biological Sciences, University of California, San Diego, La Jolla, California, United States of America; 2 Department of Immunology, Duke University, Durham, North Carolina, United Stated of America; SRUC: Scotland’s Rural College, UNITED KINGDOM

## Abstract

The threat to public health posed by drug-resistant bacteria is rapidly increasing, as some of healthcare’s most potent antibiotics are becoming obsolete. Approximately two-thirds of the world’s antibiotics are derived from natural products produced by *Streptomyces* encoded biosynthetic gene clusters. Thus, to identify novel gene clusters, we sequenced the genomes of four bioactive *Streptomyces* strains isolated from the soil in San Diego County and used Bacterial Cytological Profiling adapted for agar plate culturing in order to examine the mechanisms of bacterial inhibition exhibited by these strains. In the four strains, we identified 104 biosynthetic gene clusters. Some of these clusters were predicted to produce previously studied antibiotics; however, the known mechanisms of these molecules could not fully account for the antibacterial activity exhibited by the strains, suggesting that novel clusters might encode antibiotics. When assessed for their ability to inhibit the growth of clinically isolated pathogens, three *Streptomyces* strains demonstrated activity against methicillin-resistant *Staphylococcus aureus*. Additionally, due to the utility of bacteriophages for genetically manipulating bacterial strains via transduction, we also isolated four new phages (BartholomewSD, IceWarrior, Shawty, and TrvxScott) against *S*. *platensis*. A genomic analysis of our phages revealed nearly 200 uncharacterized proteins, including a new site-specific serine integrase that could prove to be a useful genetic tool. Sequence analysis of the *Streptomyces* strains identified CRISPR-Cas systems and specific spacer sequences that allowed us to predict phage host ranges. Ultimately, this study identified *Streptomyces* strains with the potential to produce novel chemical matter as well as integrase-encoding phages that could potentially be used to manipulate these strains.

## Introduction

Antibiotic discovery is an international priority requiring immediate action [1]. The increasing prevalence of multi-drug resistant (MDR) bacterial pathogens has resulted in an increased use of last-resort antibiotics [1–3]. Microbes that produce natural products are the most prolific source of clinically approved antibiotics [4]. Soil dwelling Actinobacteria, notably *Streptomyces*, account for two-thirds of the antibiotics currently on the market [5–7]. Despite intensive studies, however, the full potential of microbes to produce natural products has not been realized [8]. Genome mining studies have shown that microbes encode many biosynthetic gene clusters (BGCs) that have not yet been characterized [8]. It is widely assumed that many of these clusters produce novel natural products and that further characterization of *Streptomyces* bacteria increases the probability of identifying molecules with unique chemical structures and new mechanisms of action [9].

In addition to identifying *Streptomyces* strains containing potentially novel BGCs, it is necessary to improve on the conventional approaches used in natural product antibiotic discovery. One of the major stumbling blocks in natural product discovery is dereplication since the isolation of bioactive molecules often yields antibiotics that have previously been discovered [10]. We recently developed Bacterial Cytological Profiling (BCP) as a new whole-cell screening technique that can be used to rapidly identify the mechanism of action (MOA) of antibiotics [11–16]. BCP can accurately identify the pathway inhibited by antibacterial compounds present in unfractionated crude organic extracts and can be used to guide the purification of molecules with specific bioactivities [11, 15]. BCP can also be used to screen bacterial strains directly on petri plates to identify and prioritize those strains that produce molecules with desired MOAs [15]. In effect, BCP helps with the problem of dereplication by allowing for the determination of the MOA of antibiotics synthesized by a particular *Streptomyces* strain before labor-intensive antibiotic purification efforts are performed.

Since many BGCs are not expressed under laboratory conditions, genetic methods are often used to augment their expression and facilitate the identification and purification of their products [17]. Sometimes, increased expression can be achieved using techniques such as CRISPR/Cas or plasmid cloning and overexpression [17]. However, there is still an occasional need to move large chromosomal regions from one strain to another via transduction to engineer strains optimally suited for antibiotic production. Transduction requires a phage capable of infecting the strain(s) of interest. Moreover, because phages generally display narrow host ranges [18] and relatively few *Streptomyces* phages have been isolated [19] compared to the large number of studied *Streptomyces* bacteria [20], phages aptly suited for genetic manipulations are not available for the majority of antibiotic producing *Streptomyces* strains isolated. In addition, phage derived enzymes such as recombinases and integrases can also be used to engineer new strains [21–25]. Thus, studying the phages that infect antibiotic-producing *Streptomyces* strains could not only yield new transducing phages but potentially also new genetic tools for strain engineering.

Here we describe the isolation and characterization of *Streptomyces* strains and phages. We used a combination of bioinformatics and BCP to characterize the antibiotic biosynthetic potential of four *Streptomyces* strains that displayed an ability to inhibit Gram-negative and Gram-positive bacterial growth. Additionally, we isolated four new phages and assessed their abilities to infect our *Streptomyces* strains, which contained many CRISPRs. The proteins encoded by the phages were subjected to bioinformatic analyses to identify putative integrases that might be used for genetic manipulations. This work highlights a novel set of gene clusters and *Streptomyces* sp. phages that serve as a starting point for the isolation of potentially novel natural products.

## Results and discussion

### Isolation and antibacterial activities of *Streptomyces* sp.

**Fig 1:**
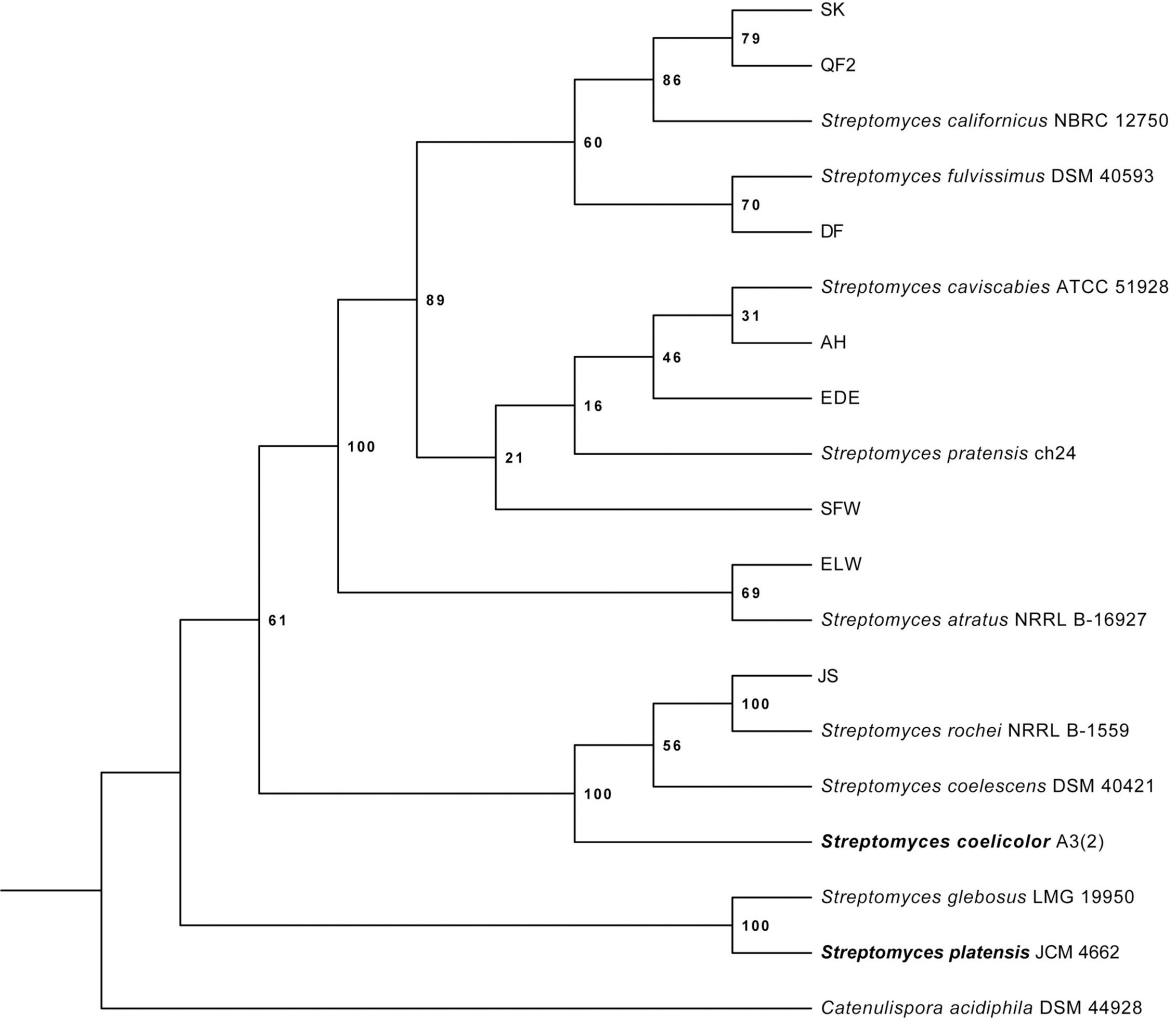
The maximum likelihood phylogeny of *Streptomyces* bacteria isolated from soil samples. This phylogenetic tree was constructed by aligning PCR-amplified 16S rRNA sequences with MUSCLE and analyzing with RAxML. Laboratory strains A3(2) and JCM 4662 (in bold) and all type strains are named.

**Fig 2:**
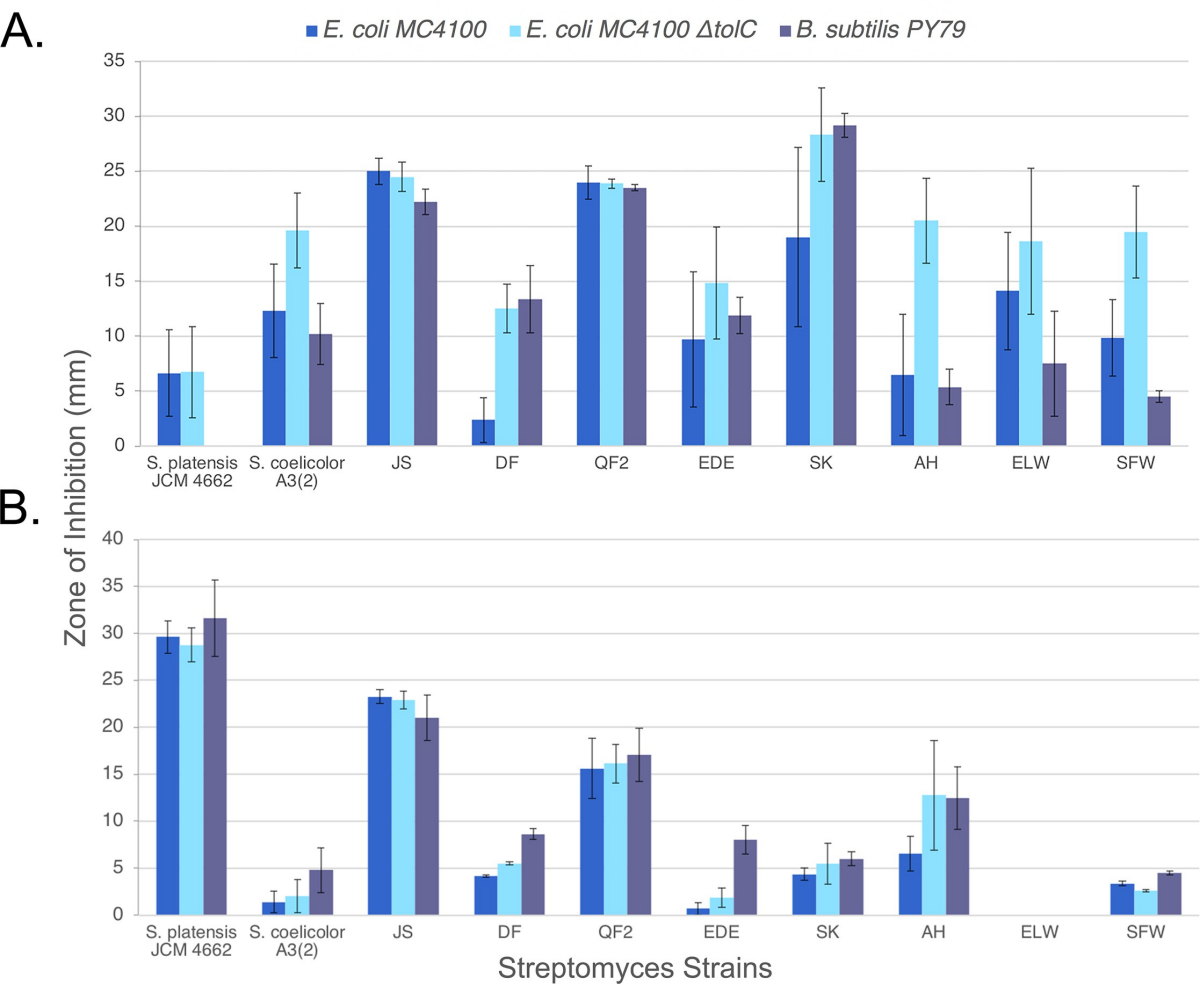
Inhibition of bacterial growth by *Streptomyces* isolates. The cross-streak method was used to measure the zone of inhibition among ten *Streptomyces* strains against two Gram-negative *E*. *coli* strains (MC4100 WT, JP313 Δ*tolC*), and one Gram-positive strain *B*. *subtilis* PY79 on (**A**) LB and (**B**) AIA. Error bars represent the standard deviation of three independent trials.

To identify *Streptomyces* strains containing potentially novel BGCs, we collected 28 unique soil samples from sites across San Diego County. From these samples, we isolated a total of eight bacterial strains based on colony morphology. The genus level classification of the eight isolates was confirmed as *Streptomyces* using the phylogeny of their 16S rRNA sequences as well as data from type strains (Fig 1 and Table 1). Each of the strains isolated in this study were part of a well-supported clade including at least one type strain, These strains (designated JS, DF, QF2, EDE, SK, AH, ELW, and SFW) and two known species (*Streptomyces coelicolor* A3(2) and *Streptomyces platensis* AB045882) were screened using the cross-streak method for their ability to inhibit the growth of wild type *E*. *coli* MC4100, an efflux defective mutant *E*. *coli* JP313 Δ*tolC*, and *B*. *subtilis* PY79. Since the production of bioactive secondary metabolites is highly dependent on growth conditions, this screen was conducted on actinomycete isolation agar (AIA) as well as Luria Broth (LB) agar. Each of the 10 strains proved capable of inhibiting the growth of *E*. *coli* and/or *B*. *subtilis* on at least one of the tested media (Fig 2), suggesting that these strains likely produce antibiotics. As expected, however, the production of antibiotics often depended upon whether the strain was grown on AIA or LB agar. For example, strain ELW was incapable of inhibiting the growth of Gram-negative and Gram-positive bacteria when grown on AIA. However, when grown on LB agar, strain ELW inhibited the growth of both Gram-negative and Gram-positive bacteria. Conversely, strains JS and QF2 exhibited growth inhibition regardless of the media on which they were grown.

**Table 1:** Top NCBI BlastN hits of the 16S rRNA gene sequences.

Sample ID	NCBI BlastN Top 16S ribosomal RNA Hit Description	Max Score	Total Score	Query Cover	E value	Percent Identity	Accession No.
*S*. *platensis* JCM 4662	*Streptomyces platensis* strain JCM 4662	2748	2748	100%	0	100%	NR_024761.1
*S*. *coelicolor* A3(2)	*Streptomyces coelescens* strain AS 4.1594	2793	2793	98%	0	99.93%	NR_027222.1
JS	*Streptomyces rochei* strain NRRL B-1559	2741	2741	91%	0	99.93%	NR_116078.1
DF	*Streptomyces fulvissimus* strain DSM 40593	2691	2691	100%	0	99.93%	NR_103947.1
QF2	*Streptomyces californicus* strain NBRC 12750	2699	2699	100%	0	100%	NR_112257.1
EDE	*Streptomyces pratensis* strain ch24	1238	1238	99%	0	98.99%	NR_125616.1
SK	*Streptomyces californicus* strain NBRC 12750	1242	1242	100%	0	100%	NR_112257.1
AH	*Streptomyces pratensis* strain ch24	1194	1194	100%	0	100%	NR_125616.1
ELW	*Streptomyces atratus* strain NRRL B-16927	1138	1138	100%	0	99.84%	NR_043490.1
SFW	*Streptomyces caviscabies* strain ATCC 51928	2767	2767	100%	0	99.87%	NR_114493.1

### Mechanistic analysis of natural products produced by four *Streptomyces* isolates

**Fig 3:**
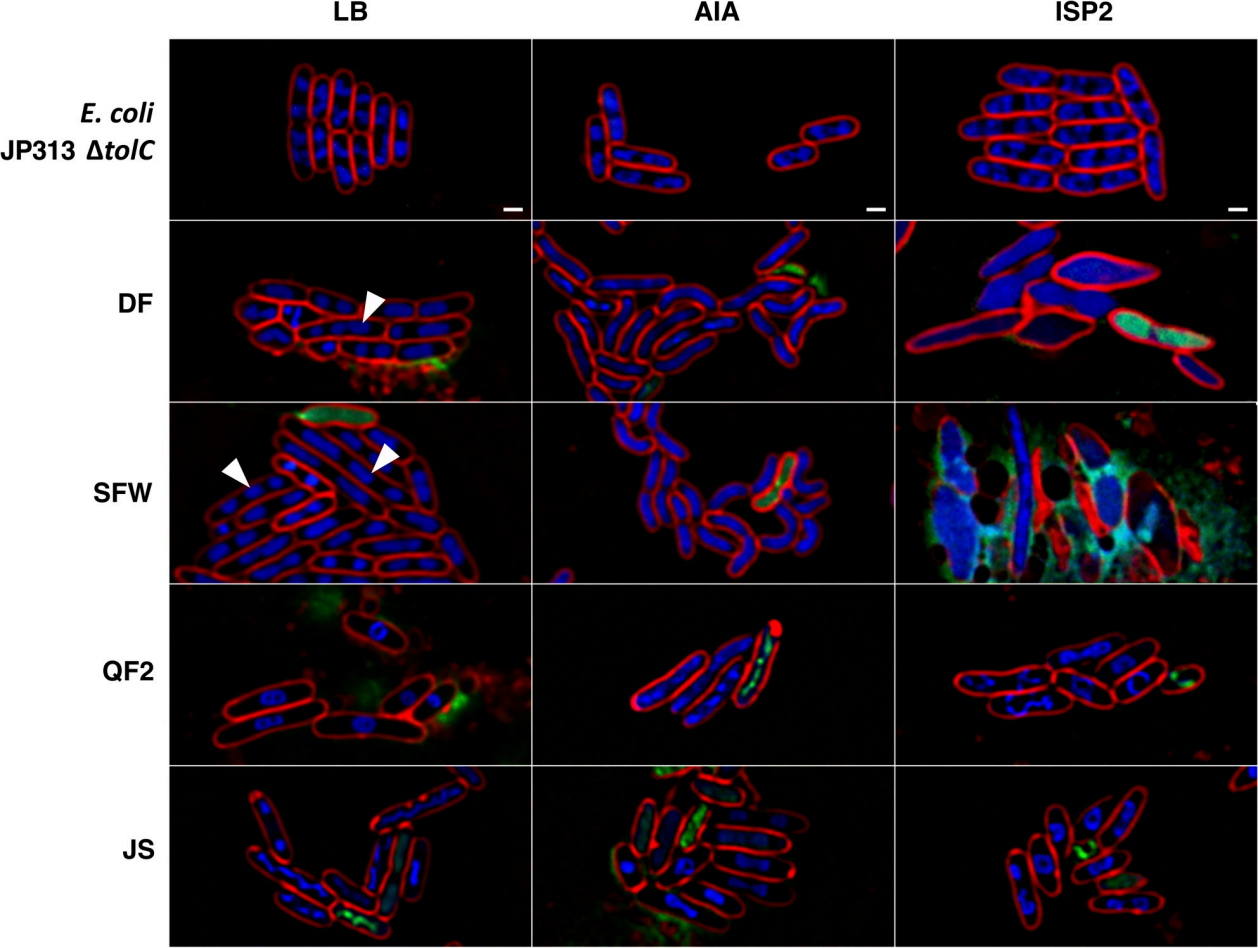
BCP phenotypes of *E*. *coli* JP313 Δ*tolC* exposed to natural products produced by four *Streptomyces* soil isolates grown on different solid media. Also displayed, are *E*. *coli* JP313 Δ*tolC* untreated controls grown on the tested media (LB agar, AIA, and ISP2 agar). White arrows indicate cells with three chromosomes. BCP images were collected after staining the cells with FM4-64 (red), DAPI (blue), and SYTOX-green (green). The scale bar represents one micron.

Strains QF2, JS, SFW and DF all inhibited the growth of *E*. *coli* Δ*tolC* when grown on either AIA or LB agar, but in each case, the mechanism underlying inhibition was unknown. Thus, we utilized BCP to examine the mechanism of growth inhibition exhibited by the antibacterial natural products synthesized by these four *Streptomyces* isolates. Each of the four strains was grown on three different media: LB, AIA, or International *Streptomyces* Project-2 media (ISP2) for 5 days to allow for the synthesis and excretion of natural products into the surrounding agar. We then added exponentially growing *E*. *coli* cells adjacent to the *Streptomyces* lawn. After two hours of incubation at 30°C, the *E*. *coli* cells were stained with fluorescent dyes and imaged with high resolution fluorescence microscopy. *E*. *coli* cells incubated adjacent to each of the four *Streptomyces* isolates displayed characteristic cytological profiles that, in some cases, allowed for the classification of these strains according to the MOA of the natural products they produced (Fig 3).

When grown on either LB or ISP2, strain QF2 synthesized an antibiotic that caused the DNA of affected *E*. *coli* cells to assume a toroidal conformation (Fig 3). This phenotype is characteristic of bacteria treated with protein synthesis inhibitors such as chloramphenicol [11, 26], and thus, we concluded that strain QF2 can synthesize a translation-inhibiting natural product. QF2 also produced a membrane-active secondary metabolite, evidenced by visible membrane abnormalities as well as Sytox Green permeability under all tested nutrient conditions (Fig 3). Strain JS appeared to induce similar phenotypes in *E*. *coli*, though under different growth conditions; protein synthesis inhibition phenotypes were observed on AIA and ISP2 but not on LB. Similar to strain QF2, Sytox Green permeability was observed in some cells regardless of the medium on which strain JS was grown.

Strain SFW induced distinct phenotypes in *E*. *coli* cells under each of the three nutrient conditions (Fig 3). On LB, a significant number of *E*. *coli* cells grown in the presence of strain SFW appeared to contain three chromosomes (white arrows), a phenotype that was not present in the untreated control cells. When strain SFW was grown on AIA, the *E*. *coli* cells became bent and lost their characteristic rod shape. Finally, strain SFW grown on ISP2 induced substantial swelling in *E*. *coli* cells that ultimately led to lysis. Notably, *E*. *coli* cells grown in the presence of strain DF exhibited nearly these same phenotypes under identical growth conditions, suggesting that these two strains produce compounds targeting similar pathways.

### Genomic analysis of four *Streptomyces* isolates

**Fig 4:**
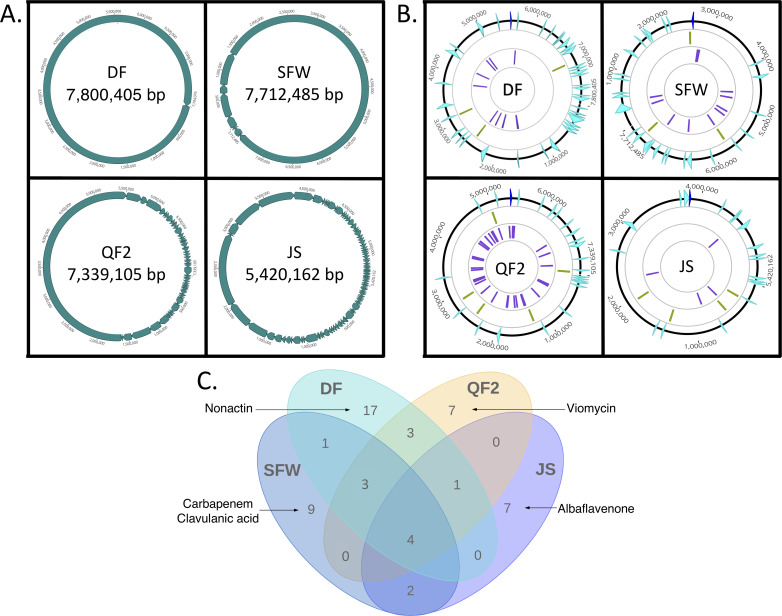
Genome characteristics of *Streptomyces* strains DF, SFW, QF2, and JS isolated from soil samples. (**A**) Circularized representations of the linear genomes of the four bacterial isolates displayed as assembled contigs obtained from genome sequencing. (**B**) Genomic annotations are displayed on separate tracks; from outermost to innermost, genomes are oriented according to their threonine operons (dark blue). Predicted biosynthetic gene clusters (light blue), loci of Cas-associated protein-coding genes (green), and CRISPR arrays (purple) are shown. (**C**) A Venn diagram displaying the numbers of BGCs that are shared by and unique to the genomes of our isolates. Five clusters of particular importance are explicitly named.

To better understand how strains QF2, JS, SFW and DF inhibited bacterial growth, we sequenced their genomes and aligned them to the most similar genomes in the NCBI database (Fig 4A). Sequence reads for strain DF were assembled into a single contig that was most similar to the genome of *S*. *fulvissimus* DSM 40593. Sequencing of strains QF2, JS, and SFW yielded multiple contigs that were aligned to the genomes of *S*. *globisporus* C-1027, *S*. *parvulus* 12434, and *S*. *pratensis* ATCC 33331, respectively.

**Fig 5:**
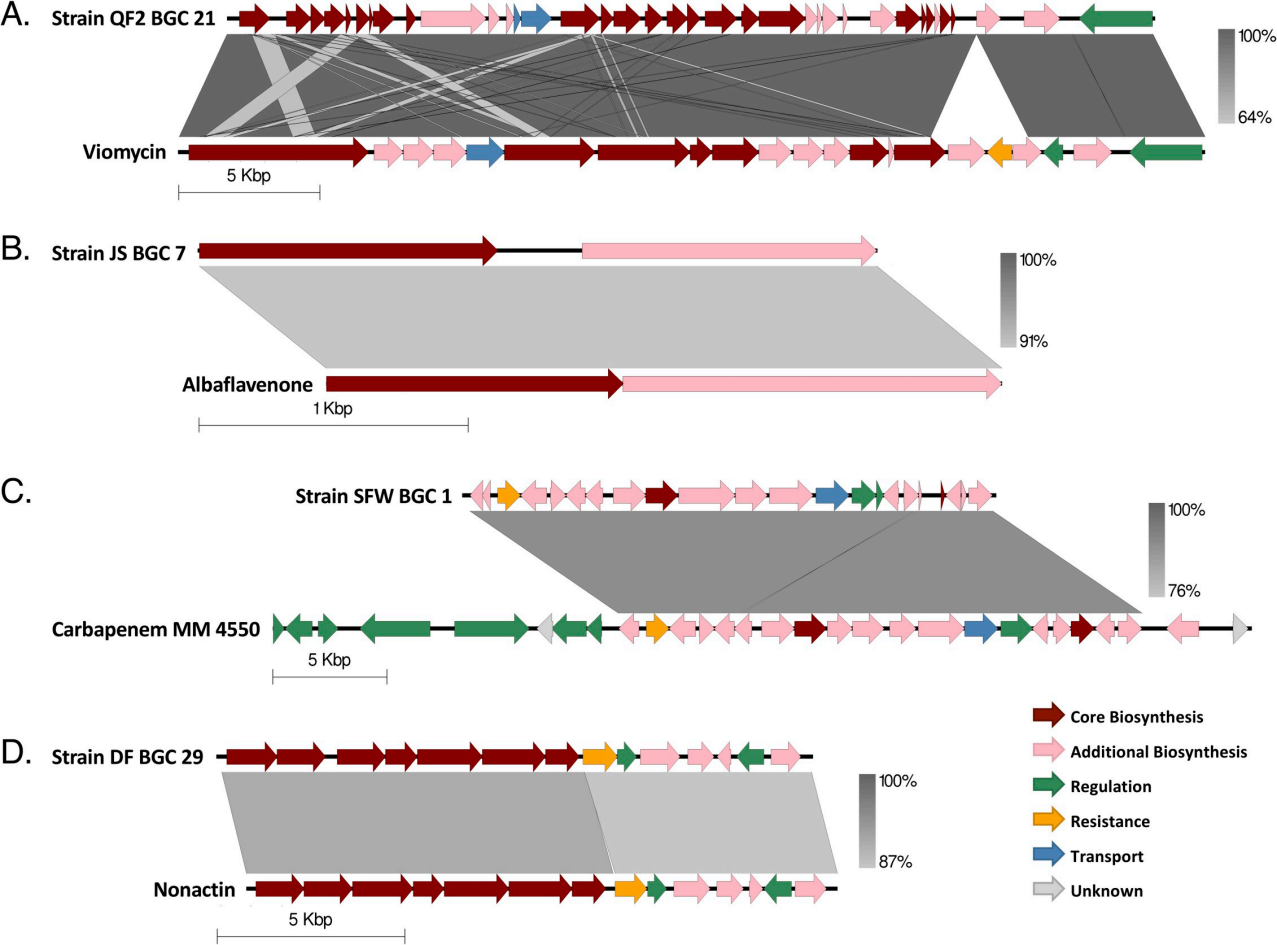
Comparison of BGCs encoded in the genomes of bacterial soil isolates and the predicted most similar, previously characterized BGC with an antibacterial product. (**A**) Strain QF2, BGC 21, compared to the BGC previously described to produce the antibiotic viomycin (NCBI Acc No. AY263398.1), encoded in the WGS of *S*. *vinaceus* ATCC 11861. (**B**) Strain JS, BGC 7, compared to the BGC previously described to produce the antibacterial sesquiterpene Albaflavenone (NCBI Acc No. AL645882.2), encoded in the WGS of *S*. *coelicolor* A3(2). (**C**) Strain SFW, BGC 1, compared to the BGC previously described to produce the antibacterial beta-lactam Carbapenem MM 4550 (NCBI Acc No. KF042303.1), encoded in the WGS of *S*. *argenteolus* ATCC 11009. (**D**) Strain DF, BGC 29, compared to the BGC previously described to produce the ammonium ionophore antibiotic Nonactin (NCBI Acc No. AF074603.2), encoded in the WGS of *S*. *griseus subsp*. *griseus* ETH A7796. Cluster comparisons were constructed in Easyfig. Regions of nucleotide homology are indicated on a gray scale and genes are colored according to the putative function of the corresponding protein product.

In order to identify predicted gene clusters associated with secondary metabolism, the assembled genome sequences for strains QF2, JS, SFW and DF were annotated using RASTtk [27] and submitted to AntiSMASH 5.0 [28] (Fig 4B). Each strain encoded between 18 and 37 BGCs, some of which were present in multiple strains (Fig 4C). Additionally, some of the encoded clusters closely resembled known BGCs in the MIBiG repository [29]. For example, of the 23 putative BGCs identified in the genome of strain QF2 (Table 2), one of them (cluster 21) was similar to the viomycin BGC (Fig 5A). Viomycin inhibits protein synthesis by stabilizing tRNAs in the A site of the bacterial ribosome, inhibiting translocation [30]. According to AntiSMASH, 66% of the genes within the viomycin BGC were similar to genes within cluster 21. However, a global pairwise alignment of cluster 21 and the viomycin BGC revealed that the nucleotide sequence of cluster 21 is actually 98.5% identical over 32.5kb of the 36kb viomycin BGC (Fig 5A). This suggests that a viomycin related molecule is synthesized by strain QF2 and may account for strain’s ability to inhibit protein synthesis in *E*. *coli* (Fig 3). While some of the other clusters in the QF2 genome (Table 2: clusters 2, 4, 7, 8, 9, 12, 13, 22) have significant similarity to known BGCs, no other clusters appear to produce known antibiotics.

**Table 2:** BGCs encoded within the draft genome sequence of strain QF2.

Strain—QF2							
Cluster	Type	Most Similar MiBIG Cluster and Predicted Percent Similarity	Antibacterial Activity	MIBig BGC-ID	Minimum	Maximum	Length (nt.)
1	butyrolactone	Coelimycin P1 T1PKS (8%)		BGC0000038	28795	39496	10702
2	terpene	Geosmin Terpene (100%)		BGC0001181	60346	82527	22182
3	NRPS	Griseobactin NRPS (35%)		BGC0000368	101394	123548	22155
4	NRPS	Coelichelin NRPS (72%)		BGC0000325	123549	145157	21609
5	T3PKS	Herboxidiene T1PKS, T3PKS (6%)		BGC0001065	184513	202853	18341
6	terpene	Isorenieratene Terpene (57%)		BGC0000664	681001	697369	16369
7	ectoine	Ectoine Other (75%)		BGC0000853	1125565	1134445	8881
8	T2PKS	Griseorhodin T2PKS (69%)		BGC0000230	1908342	1950906	42565
9	siderophore	Desferrioxamine B Siderophore (80%)		BGC0000941	2278919	2290697	11779
10	LAP,thiopeptide	-	-	-	2690041	2722548	32508
11	ectoine,butyrolactone	Showdomycin Other (47%)	Nucleic Acid and Protein Synthesis	BGC0001778	3344033	3359401	15369
12	melanin	Melanin Other (100%)		BGC0000911	4777563	4787988	10426
13	lanthipeptide	AmfS Lanthipeptide (100%)		BGC0000496	5105643	5127766	22124
14	terpene	-	-	-	5476141	5497007	20867
15	siderophore	Ficellomycin NRPS (3%)	DNA Replication	BGC0001593	5881146	5896034	14889
16	NRPS	Vioprolide A NRPS (25%)		BGC0001822	6093895	6137611	43717
17	bacteriocin	-	-	-	6194201	6205608	11408
18	NRPS-like	-	-	-	6430967	6442494	11528
19	NRPS-like,ladderane,arylpolyene	Skyllamycin NRPS (14%)	Unknown MOA	BGC0000429	6608620	6645073	36454
20	terpene	Hopene Terpene (46%)		BGC0000663	6755130	6764031	8902
21	NRPS,T1PKS	Viomycin NRPS (66%)	Protein Synthesis	BGC0000458	6806327	6870853	64527
22	T3PKS	Alkylresorcinol T3PKS (66%)	Unknown MOA	BGC0000282	7058484	7080991	22508
23	lassopeptide	-	-	-	7260793	7282889	22097

The most similar BGCs in the MIBiG database are listed, as well as the percentage of genes in each MIBiG known cluster that have similarity to genes in the corresponding QF2 cluster. In cases where the most similar known BGC produces an antibiotic, the MOA was listed (Showdomycin [31], Ficellomycin [32], Skyllamycin [33], Viomycin [30], Alkylresorcinol [34]).

Strain JS contained 18 putative BGCs, six of which shared significant similarity (>60% of genes in common) with a known cluster (Table 3). Of these six, however, only cluster 7 was predicted to produce an antibiotic. All of the genes constituting a known terpene cluster that produces albaflavenone were present in cluster 7 (Fig 5B). Albalfavenone is capable of inhibiting the growth of *B*. *subtilis* by an unknown MOA [35] and has previously been isolated from *S*. *coelicolor* A3(2) [36], a close relative of strain JS. Since the MOA of albaflavenone is unknown, it’s not clear whether the products of cluster 7 or of a different cluster are responsible for the inhibition of protein synthesis and/or the membrane permeability observed in *E*. *coli* (Fig 3).

**Table 3:** BGCs encoded within the draft genome sequence of strain JS.

Strain—JS							
Cluster	Type	Most Similar MiBIG Cluster and Predicted Percent Similarity	Antibacterial Activity	MIBig BGC-ID	Minimum	Maximum	Length (nt.)
1	T3PKS	Herboxidiene T1PKS, T3PKS (7%)		BGC0001065	214109	229085	14977
2	ectoine	Ectoine Other (100%)		BGC0000853	672467	682865	10399
3	melanin	Melanin Other (60%)		BGC0000911	1414668	1425276	10609
4	siderophore	Desferrioxamine B Siderophore (66%)		BGC0000941	1507497	1519344	11848
5	furan	Methylenomycin Other (9%)	Potentially Inhibits Cell Wall Biosynthesis	BGC0000914	2667706	2688702	20997
6	NRPS	Ansamitocin P-3 T1PKS (7%)		BGC0001511	3021294	3076234	54941
7	terpene	Albaflavenone Terpene (100%)	Unknown MOA	BGC0000660	3743916	3764845	20930
8	T2PKS	Spore pigment T2PKS (66%)		BGC0000271	3800219	3857023	56805
9	siderophore	-	-	-	4274698	4286154	11457
10	bacteriocin	-	-	-	4471015	4482384	11370
11	terpene	-	-	-	4488880	4508472	19593
12	NRPS	Lipopeptide 8D1-1 & 8D1-2 NRPS (25%)	PMF Collapse	BGC0001370	4616241	4669719	53479
13	NRPS	Lipopeptide 8D1-1 & 8D1-2 NRPS (15%)	PMF Collapse	BGC0001370	4929292	4967094	37803
14	terpene	Hopene Terpene (76%)		BGC0000663	5042481	5069167	26687
15	terpene	Lysolipin T2PKS (4%)	Cell Wall Biosynthesis	BGC0000242	5088579	5103665	15087
16	T1PKS	Candicidin T1PKS (28%)		BGC0000034	5331251	5350185	18935
17	T2PKS,butyrolactone	Kinamycin T2PKS (25%)	DNA Synthesis	BGC0000236	5370217	5395391	25175
18	T1PKS	FR-008/Levorin A3 T1PKS (28%)		BGC0000061	5395392	5413565	18174

The most similar BGCs in the MIBiG database are listed, as well as the percentage of genes in each MIBiG known cluster that have similarity to genes in the corresponding JS cluster. In cases where the most similar known BGC produces an antibiotic, the MOA was listed (Methylenomycin [37], Albaflavenone [35], Lipopeptide 8D1-1 & 8D1-2 [38], Lysolipin [39, 40]).

Of the 26 putative BGCs that were identified in the genome of strain SFW, only one cluster shared a high percentage of genes in common with a known antibiotic-producing cluster (Table 4). Cluster 1 shared similarity with 62% of the genes within a known BGC that produces carbapenems (Fig 5C), a class of beta-lactam antibiotics that inhibit cell wall biogenesis [41, 42]. Additionally, cluster 4 contained a low percentage of genes in common with a BGC involved in the synthesis of clavulanic acid, which inhibits beta-lactamase and consequently strengthens the bactericidal activity of beta-lactams. Cluster 1 (and perhaps cluster 4) could, therefore, contribute to the synthesis of bioactive molecules that account for the inhibition of *E*. *coli* cell wall biogenesis on ISP2 media (Fig 3).

**Table 4:** BGCs encoded within the draft genome sequence of strain SFW.

Strain—SFW							
Cluster	Type	Most Similar MiBIG Cluster and Predicted Percent Similarity	Antibacterial Activity	MIBig BGC-ID	Minimum	Maximum	Length (nt.)
1	NRPS, blactam	Carbapenem MM 4550 Other (62%)	Cell Wall Biosynthesis	BGC0000842	280243	420495	140253
2	NRPS	Coelichelin NRPS (90%)		BGC0000325	537068	587954	50887
3	terpene	Isorenieratene Terpene (28%)		BGC0000664	601924	615201	13278
4	blactam	Clavulanic acid Other (20%)	Beta-lactamase Inhibition	BGC0000845	839861	863248	23388
5	terpene	Hopene Terpene (69%)		BGC0000663	920123	946635	26513
6	T1PKS	Sceliphrolactam T1PKS (72%)		BGC0001770	1325302	1388343	63042
7	bacteriocin	-	-	-	1598693	1609196	10504
8	lanthipeptide	Kanamycin Saccharide (1%)	Protein Synthesis	BGC0000703	1734703	1760347	25645
9	NRPS	Lipopeptide 8D1-1 & 8D1-2 NRPS (6%)	PMF Collapse	BGC0001370	1773501	1830128	56628
10	siderophore	Ficellomycin NRPS (3%)	DNA replication	BGC0001593	2107372	2120491	13120
11	terpene	-	-	-	2186197	2205874	19678
12	butyrolactone	Lactonamycin T2PKS (3%)	Protein Synthesis	BGC0000238	4018281	4029096	10816
13	NRPS	Istamycin Saccharide (11%)	Protein Synthesis	BGC0000700	4251191	4307412	56222
14	siderophore	Desferrioxamine B Siderophore (83%)		BGC0000941	4924798	4936579	11782
15	lanthipeptide	-	-	-	5321361	5346350	24990
16	terpene	-	-	-	5588840	5608518	19679
17	ectoine	Ectoine Other (100%)		BGC0000853	6072388	6080990	8603
18	T2PKS, PKS-like	Cinerubin B T2PKS (25%)	DNA Intercalation	BGC0000212	6443938	6515214	71277
19	terpene	Steffimycin T2PKS-Saccharide (16%)		BGC0000273	6560271	6580717	20447
20	terpene, ectoine	Ectoine Other (100%)		BGC0000853	6860752	6881669	20918
21	bacteriocin	-	-	-	6909859	6920014	10156
22	T3PKS	Tetronasin T1PKS (11%)	PMF Collapse	BGC0000163	7071589	7112647	41059
23	melanin	Melanin Other (100%)		BGC0000911	7208921	7219385	10465
24	T2PKS, terpene	Spore pigment T2PKS (83%)		BGC0000271	7244899	7317424	72526
25	NRPS	Rimosamide NRPS (21%)		BGC0001760	7458615	7511513	52899
26	butyrolactone	-	-	-	7592249	7602533	10285

The most similar BGCs in the MIBiG database are listed, as well as the percentage of genes in each MIBiG known cluster that have similarity to genes in the corresponding SFW cluster. In cases where the most similar known BGC produces an antibiotic, the MOA was listed (Carbapenem [42], Clavulanic acid [43], Kanamycin [44], Lipopeptide 8D1-1 & 8D1-2 [38], Ficellomycin [32], Lactonamycin [45, 46], Istamycin [47–49], Cinerubin [50], Tetronasin [51]).

Strain DF encoded 37 BGCs (Table 5). Despite this rich supply of BGCs, however, we were only able to identify one cluster that likely participates in the synthesis of an antibiotic with a confirmed MOA. According to AntiSMASH v5.0, cluster 29 shared 92% gene identity with a known BGC that produces nonactin, a bioactive ionophore that disrupts membrane potential [52] (Fig 5D). The known clusters could not fully account for the antibacterial activity exhibited by strain DF (Fig 3), suggesting that antibiotics might be produced by novel clusters.

**Table 5:** BGCs encoded within the closed genome sequence of strain DF.

Strain—DF							
Cluster	Type	Most Similar MiBIG Cluster and Predicted Percent Similarity	Antibacterial Activity	MIBig BGC-ID	Minimum	Maximum	Length (nt.)
1	ectoine	-	-	-	56852	65619	8767
2	butyrolactone	Coelimycin P1 T1PKS (16%)		BGC0000038	158609	168403	9794
3	terpene	Geosmin Terpene (100%)		BGC0001181	198747	220149	21402
4	transAT-PKS, PKS-like,T1PKS, NRPS	Streptobactin NRPS (76%)		BGC0000368	227951	344372	116421
5	NRPS	Coelichelin NRPS (81%)		BGC0000325	365595	413819	48224
6	NRPS, T1PKS	Arsenopolyketides Other (45%)	Unknown MOA	BGC0001283	423679	473545	49866
7	T3PKS	Herboxidiene PKS (6%)		BGC0001065	485172	524155	38983
8	T2PKS	Hiroshidine PKS (41%)	Unknown MOA	BGC0001960	862740	934422	71682
9	terpene	Steffimycin D T2PKS-Saccharide (19%)		BGC0000273	1083717	1102744	19027
10	ectoine	Ectoine Other (100%)		BGC0000853	1581580	1591978	10398
11	NRPS, PKS-like	Decaplanin NRPS (7%)	Cell Wall	BGC0001459	2187015	2263126	76111
12	lanthipeptide	-	-	-	2620147	2641946	21799
13	siderophore	Desferrioxamine B Siderophore (100%)		BGC0000941	2701211	2711611	10400
14	NRPS-like	Bottromycin A2 RiPP (39%)	Protein Synthesis	BGC0000469	2808481	2851819	43338
15	thiopeptide, LAP	-	-	-	3106169	3139358	33189
16	NRPS	Phosphonoglycans Saccharide (3%)		BGC0000806	3332988	3396052	63064
17	betalactone	Divergolide A-D T1PKS (6%)		BGC0001119	3744384	3772058	27674
18	T2PKS	Prejadomycin, Rabelomycin, Gaudimycin A, C-D, & UWM6 T2PKS-Saccharide (27%)	Unknown MOA	BGC0000262	4307584	4380138	72554
19	lassopeptide	Keywimycin RiPP (100%)		BGC0001634	4424144	4446763	22619
20	T1PKS	Argimycin PI-II, IV-VI, IX & Nigrifactin T1PKS (29%)		BGC0001433	4982495	5043259	60764
21	lanthipeptide	AmfS Lanthipeptide (100%)		BGC0000496	5322352	5345015	22663
22	terpene	-	-	-	5682792	5697907	15115
23	siderophore	Ficellomycin NRPS (3%)	DNA replication	BGC0001593	6155222	6169824	14602
24	butyrolactone	-	-	-	6322803	6333756	10953
25	bacteriocin	-	-	-	6494268	6503925	9657
26	terpene	2-methylisoborneol Terpene (100%)		BGC0000658	6519618	6539195	19577
27	NRPS	Asukamycin T2PKS (12%)	Unknown MOA	BGC0000187	6625892	6684302	58410
28	NRPS-like, arylpolyene	Formicamycins A-M PKS (11%)	Unknown MOA	BGC0001590	6729705	6772824	43119
29	NRPS	Nonactin T2PKS (92%)	Dissipates Transmembrane Electric Potential	BGC0000252	6780952	6844946	63994
30	terpene	Hopene Terpene (69%)		BGC0000663	7099119	7125294	26175
31	linaridin	Pentostatine & Vidarabine Other (9%)		BGC0001735	7147103	7167711	20608
32	T1PKS, NRPS	SGR PTMs NRPS, T1PKS (100%)	Unknown MOA	BGC0001043	7205942	7253575	47633
33	bacteriocin	-	-	-	7266691	7277488	10797
34	melanin	Melanin Other (100%)		BGC0000911	7458714	7469181	10467
35	T3PKS	Herboxidiene T1PKS, T3PKS (9%)		BGC0001065	7501628	7542680	41052
36	terpene	Isorenieratene Terpene (100%)		BGC0000664	7633017	7658370	25353
37	NRPS, T1PKS, LAP, thiopeptide	Lactazole Thiopeptide (33%)		BGC0000606	7671732	7738578	66846

The most similar BGCs in the MIBiG database are listed, as well as the percentage of genes in each MIBiG known cluster that have similarity to genes in the corresponding DF cluster. In cases where the most similar known BGC produces an antibiotic, the MOA was listed (Arsenopolyketides [53], Hiroshidine [54], Decaplanin [55], Bottormycin [56, 57], Prejadomycin, Rabelomycin, Gaudimycin A, C-D, & UWM6 [58], Ficellomycin [59], Asukamycin [60], Formicamycins [61], Nonactin [52], SGR PTMs NRPS [62]).

### Antimicrobial activity of four *Streptomyces* isolates against clinically relevant pathogens

To assess the relevance of antibiotics produced by strains JS, DF, SFW, and QF2, we screened their ability to inhibit the growth of three clinically isolated pathogens using the cross-streak method (Table 6). Both strain QF2 and strain JS inhibited the growth of methicillin-resistant *S*. *aureus* (MRSA) and efflux-deficient *P*. *aeruginosa* PA01. These strains did not, however, inhibit the growth of the wild-type clinical isolates *P*. *aeruginosa* PA01 and *P*. *aeruginosa* P4, which were resistant to the antibiotics produced by all four *Streptomyces* isolates. Strain DF, though incapable of inhibiting the growth of *E*. *coli tolC*^*+*^ (Fig 2), did inhibit the growth of MRSA and efflux-deficient *P*. *aeruginosa* PA01. Strain SFW was the least capable of inhibiting the growth of clinical pathogens, producing antibiotics only effective against *E*. *coli tolC*^*+*^ (Fig 2).

**Table 6:** Inhibition of growth of clinically relevant pathogens by *Streptomyces* strains DF, SFW, QF2, and JS.

	Gram-Negative Bacteria					Gram-Positive Bacteria	
	*E*. *coli*		*P*. *aeruginosa*			*B*. *subtilis*	MRSA
	JP313 Δ*tolC*	MC4100	PA01	P4	PAO1 Δefflux	PY79	USA 300 TCH1516
**DF**	+	-	-	-	+	+	+
**SFW**	+	+	-	-	-	+	-
**QF2**	+	+	-	-	+	+	+
**JS**	+	+	-	-	+	+	+

Plus signs indicate growth inhibition, while minus signs indicate pathogen growth.

### Phage isolation and genome sequencing

**Fig 6:**
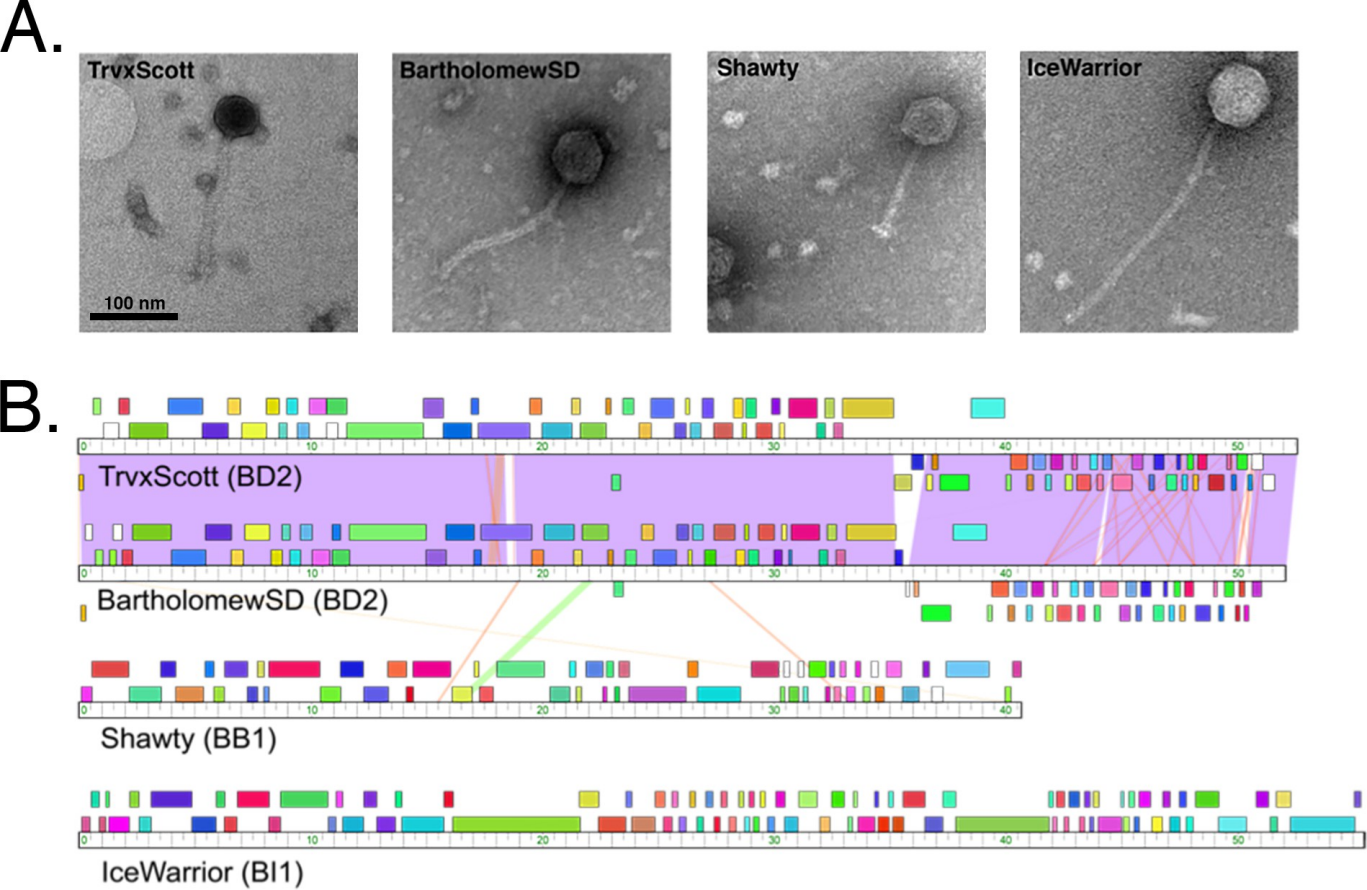
Characterization of four *Streptomyces* phages isolated from soil samples. (**A**) Electron micrographs of the four phages (IceWarrior, TrvxScott, BartholomewSD, and Shawty). Lysate samples were negatively stained and imaged with transmission electron microscopy (TEM). The scale bar represents 100 nm. (**B**) A whole-genome sequence comparison of the four phages generated by Phamerator (top to bottom: TrvxScott, BartholomewSD, Shawty, IceWarrior).

Phages capable of infecting these newly isolated *Streptomyces* strains can be used for genetic manipulation. With the goal of identifying genetic tools that could be used to augment expression of the BGCs in our bacterial isolates, we isolated bacteriophages using *S*. *platensis* as a host. This species, in particular, was chosen as a host because it is relatively well-characterized, and *S*. *platensis* phages capable of infecting our *Streptomyces* isolates could possibly be used to move (via transduction) BGCs from our isolates into a more genetically manipulatable and familiar background [63, 64]. Thus, to increase the probability that our *S*. *platensis* phages could be used for this purpose, we performed the isolation using the same soil samples from which our *Streptomyces* strains were obtained. Four *S*. *platensis* actinobacteriophages (BartholomewSD, IceWarrior, Shawty, and TrvxScott) were successfully isolated. These phages were imaged using negative-stain transmission electron microscopy (Fig 6A) and subsequently characterized as members of the family Siphoviridae due to their long filamentous tails and icosahedral capsids [65, 66]. Genome sequencing revealed that BartholomewSD (52.1 kb) and TrvxScott (52.6 kb) are 89% identical (Fig 6B) and belong to the BD2 subcluster of *Streptomyces* phages, which currently contains 20 other members [19]. IceWarrior (55.5 kb) clustered in subcluster BI1 (24 members), and Shawty (40.7 kb) clustered in BB1, a subcluster of 7 phages that includes notable members TG1 and phiC31 (Table 7) [19]. The BLASTp-predicted functions of the gene products encoded by these phages are shown in Table 8.

**Table 7:** Summary of the NCBI WGS annotations of four phage isolates.

Bacteriophage	Taxa	Genome (bp)	GC%	Genes	Cluster	Subcluster	Genbank Acc. No.
TrvxScott	unclass. Arequatrovirus	52600	67.8	81	BD	BD2	MH669016
IceWarrior	unclass. Rimavirus	55532	59.5	86	BI	BI1	MK433259
BartholomewSD	unclass. Arequatrovirus	52131	67.6	88	BD	BD2	MK460245
Shawty	unclass. Lomovskayavirus	40733	63.2	58	BB	BB1	MK433266

**Table 8:** Functions of the putative proteins encoded within the genomes of four phage isolates.

Streptomyces Phage TrvxScott taxon:2301575		Streptomyces phage Shawty taxon:2510521		Streptomyces phage IceWarrior taxon:2510515		Streptomyces phage BartholomewSD taxon:2510587	
CDS No.	Product	CDS No.	Product	CDS No.	Product	CDS No.	Product
1	hypothetical protein	1	terminase small subunit	1	hypothetical protein	1	hypothetical protein
2	HNH endonuclease	2	terminase large subunit	2	HNH endonuclease	2	hypothetical protein
3	thioredoxin	3	portal protein	3	hypothetical protein	3	HNH endonuclease
4	terminase small subunit	4	capsid maturation protease	4	hypothetical protein	4	tRNA-Phe
5	terminase large subunit	5	major capsid protein	5	endolysin	5	hypothetical protein
6	portal protein	6	head-to-tail adaptor	6	head-to-tail connector complex protein	6	thioredoxin
7	capsid maturation protease	7	hypothetical protein	7	hypothetical protein	7	hypothetical protein
8	scaffolding protein	8	major tail protein	8	terminase large subunit	8	terminase
9	major capsid protein	9	hypothetical protein	9	hypothetical protein	9	portal protein
10	head-to-tail connector complex protein	10	tail assembly chaperone	10	hypothetical protein	10	MuF-like minor capsid protein
11	head-to-tail connector complex protein	11	tail assembly chaperone	11	hypothetical protein	11	scaffolding protein
12	hypothetical protein	12	tape measure protein	12	portal protein	12	major capsid protein
13	head-to-tail connector complex protein	13	minor tail protein	13	hypothetical protein	13	head-to-tail adaptor
14	major tail protein	14	minor tail protein	14	capsid maturation protease	14	head-to-tail stopper
15	tail assembly chaperone	15	minor tail protein	15	hypothetical protein	15	hypothetical protein
16	tail assembly chaperone	16	minor tail protein	16	hypothetical protein	16	tail terminator
17	tape measure protein	17	hypothetical protein	17	major tail protein	17	major tail protein
18	minor tail protein	18	tail fiber	18	hypothetical protein	18	tail assembly chaperone
19	minor tail protein	19	lysin A	19	major tail protein	19	tail assembly chaperone
20	hypothetical protein	20	hypothetical protein	20	hypothetical protein	20	tape measure protein
21	hypothetical protein	21	deoxynucleoside monophosphate kinase	21	chitosanase	21	minor tail protein
22	hypothetical protein	22	immunity repressor	22	hypothetical protein	22	minor tail protein
23	minor tail protein	23	Cas4 family exonuclease	23	tape measure protein	23	hypothetical protein
24	hypothetical protein	24	hypothetical protein	24	minor tail protein	24	minor tail protein
25	lysin A	25	hypothetical protein	25	minor tail protein	25	hypothetical protein
26	hypothetical protein	26	hypothetical protein	26	hypothetical protein	26	hypothetical protein
27	hypothetical protein	27	hypothetical protein	27	hypothetical protein	27	hypothetical protein
28	hypothetical protein	28	hypothetical protein	28	hypothetical protein	28	lysin A
29	hypothetical protein	29	hypothetical protein	29	holin	29	hypothetical protein
30	exonuclease	30	HNH endonuclease	30	hypothetical protein	30	hypothetical protein
31	hypothetical protein	31	DNA primase	31	hypothetical protein	31	immunity repressor
32	hypothetical protein	32	restriction endonuclease	32	hypothetical protein	32	hypothetical protein
33	hypothetical protein	33	DNA polymerase I	33	hypothetical protein	33	Cas4 family exonuclease
34	deoxycytidylate deaminase	34	RNA polymerase sigma factor	34	hypothetical protein	34	hypothetical protein
35	DNA helicase	35	hypothetical protein	35	hypothetical protein	35	hypothetical protein
36	holliday junction resolvase	36	hypothetical protein	36	hypothetical protein	36	hypothetical protein
37	hypothetical protein	37	hypothetical protein	37	hypothetical protein	37	deoxycytidylate deaminase
38	DNA primase	38	hypothetical protein	38	hypothetical protein	38	DnaB-like helicase
39	DNA primase	39	hypothetical protein	39	hypothetical protein	39	holliday junction resolvase
40	hypothetical protein	40	ThyX-like thymidylate synthase	40	hypothetical protein	40	hypothetical protein
41	hypothetical protein	41	hypothetical protein	41	hypothetical protein	41	DNA primase
42	exonuclease	42	hypothetical protein	42	hypothetical protein	42	DNA primase
43	hypothetical protein	43	thioredoxin	43	hypothetical protein	43	hypothetical protein
44	HTH DNA binding protein	44	hypothetical protein	44	hypothetical protein	44	hypothetical protein
45	hypothetical protein	45	deoxycytidylate deaminase	45	hypothetical protein	45	hypothetical protein
46	ribonucleotide reductase	46	hypothetical protein	46	hypothetical protein	46	Mre11 family dsDNA break repair endo/exonuclease
47	DNA methylase	47	hypothetical protein	47	hypothetical protein	47	hypothetical protein
48	hypothetical protein	48	hypothetical protein	48	hypothetical protein	48	helix-turn-helix DNA binding protein
49	hypothetical protein	49	hypothetical protein	49	hypothetical protein	49	hypothetical protein
50	hypothetical protein	50	hypothetical protein	50	hypothetical protein	50	ribonucleotide reductase
51	HTH DNA binding protein	51	hypothetical protein	51	hypothetical protein	51	hypothetical protein
52	integrase	52	hypothetical protein	52	hypothetical protein	52	hypothetical protein
53	hypothetical protein	53	protein kinase	53	hypothetical protein	53	hypothetical protein
54	thymidylate synthase	54	integrase	54	hypothetical protein	54	hypothetical protein
55	hypothetical protein	55	tRNA-Asp	55	hypothetical protein	55	helix-turn-helix DNA binding protein
56	hypothetical protein	56	tRNA-Thr	56	hypothetical protein	56	integrase
57	hypothetical protein	57	hypothetical protein	57	hypothetical protein	57	hypothetical protein
58	hypothetical protein	58	HNH endonuclease	58	hypothetical protein	58	ThyX-like thymidylate synthase
59	hypothetical protein			59	DNA primase/polymerase	59	hypothetical protein
60	hypothetical protein			60	hypothetical protein	60	hypothetical protein
61	deoxynucleoside monophosphate kinase			61	hypothetical protein	61	hypothetical protein
62	hypothetical protein			62	hypothetical protein	62	hypothetical protein
63	hypothetical protein			63	hypothetical protein	63	hypothetical protein
64	hypothetical protein			64	hypothetical protein	64	hypothetical protein
65	hypothetical protein			65	hypothetical protein	65	deoxymononucleoside kinase
66	hypothetical protein			66	hypothetical protein	66	hypothetical protein
67	hypothetical protein			67	hypothetical protein	67	hypothetical protein
68	hypothetical protein			68	hypothetical protein	68	hypothetical protein
69	hypothetical protein			69	hypothetical protein	69	hypothetical protein
70	hypothetical protein			70	hypothetical protein	70	hypothetical protein
71	hypothetical protein			71	hypothetical protein	71	hypothetical protein
72	hypothetical protein			72	hypothetical protein	72	hypothetical protein
73	hypothetical protein			73	hypothetical protein	73	hypothetical protein
74	hypothetical protein			74	hypothetical protein	74	hypothetical protein
75	hypothetical protein			75	hypothetical protein	75	hypothetical protein
76	hypothetical protein			76	hypothetical protein	76	hypothetical protein
77	hypothetical protein			77	hypothetical protein	77	hypothetical protein
78	acetyltransferase			78	hypothetical protein	78	hypothetical protein
79	hypothetical protein			79	hypothetical protein	79	hypothetical protein
80	hypothetical protein			80	hypothetical protein	80	hypothetical protein
81	hypothetical protein			81	DNA helicase	81	hypothetical protein
				82	HNH endonuclease	82	hypothetical protein
				83	hydrolase	83	hypothetical protein
				84	DNA helicase	84	hypothetical protein
				85	helix-turn-helix DNA binding domain protein	85	hypothetical protein
				86	hypothetical protein	86	hypothetical protein
						87	hypothetical protein
						88	hypothetical protein

### Characterization of CRISPR elements in the genomes of our *Streptomyces* strains

**Fig 7:**
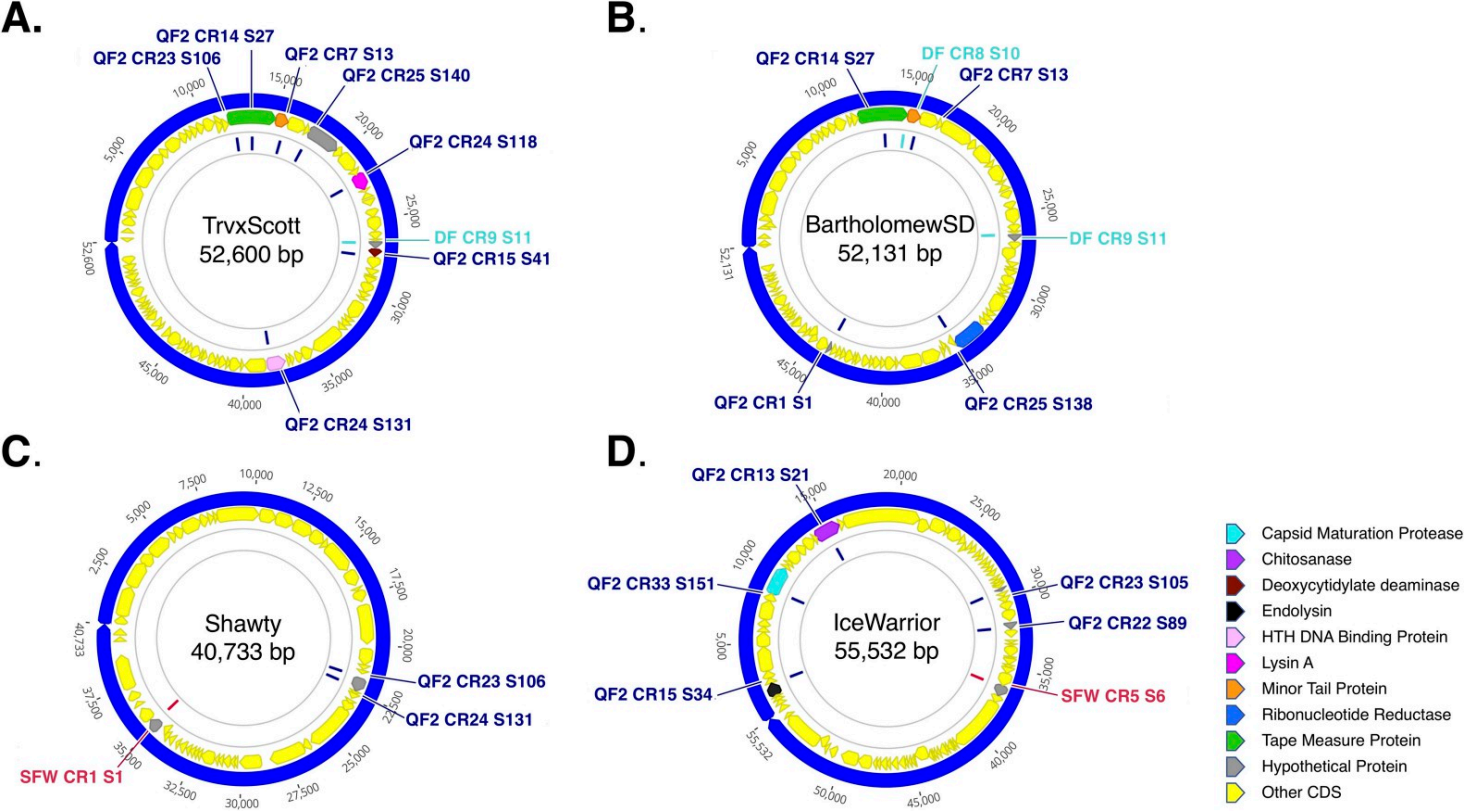
Genomic maps of phages showing regions containing sequence similarity to spacers found within the CRISPRs of strains QF2, DF, and SFW. (**A**) TrvxScott, (**B**) BartholomewSD, (**C**) Shawty, and (**D**) IceWarrior. Key displays putative functions of CRISPR targeted genes.

**Fig 8:**
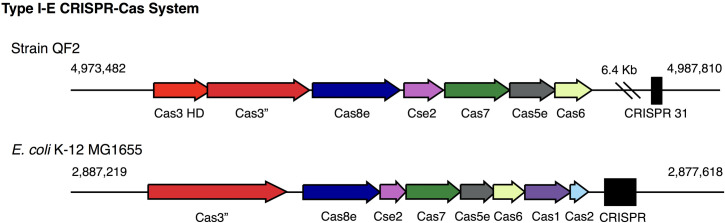
Class I, Type I-E CRISPR-Cas system encoded in the WGS of strain QF2. (**top**) The Type I-E CRISPR-Cas operon encoded by strain QG is located from 4,973,482 to 4,987,810 and includes seven genes. The Type I-E cascade is followed by CRISPR 31, consisting of two repeats and a single spacer. (**bottom**) The canonical Type I-E CRISPR-Cas system encoded in the genome of *E*. *coli* K-12 MG1655 is located from 2,887,219 to 2,877,618 and includes eight genes. The Type I-E cascade is followed by a CRISPR 31, consisting of five repeats and four spacers.

Prior to testing the ability of our phages to infect the *Streptomyces* isolates, we decided to examine the strains for complete and functional CRISPR/Cas systems. Our reasoning for this was two-fold. First, the presence of acquired spacers and their specific sequences would allow us to make predictions about whether or not our phages can infect our antibiotic-producing strains. Second, it was conceivable that in doing so we might discover a novel CRISPR/Cas-based system. Our bioinformatic analysis identified the presence of Cas enzymes and CRISPR arrays within the genomes all four of our *Streptomyces* isolates, but the abundance of CRISPRs in each strain varied greatly (Table 9). QF2 contained the largest number of predicted CRISPRs– 38 in total, scattered around the chromosome, each containing between one and 25 spacers (Fig 4B, purple; Table 10). Some predicted spacers within these arrays matched with 94–100% identity to sequences within TrvxScott (7 spacers), BartholomewSD (4 spacers), Shawty (2 spacers), and IceWarrior (5 spacers) (Tables 10 and 11). Spacers targeted a variety of genes including those encoding minor tail proteins, tape measure proteins, deoxycytidylate deaminase, helix-turn-helix DNA binding proteins, endolysin, and capsid maturation protease (Fig 7). The large number of putative spacers in the QF2 genome targeting TrvxScott, BartholomewSD, Shawty, and IceWarrior suggests that strain QF2 has likely previously encountered and acquired resistance to each of these phages. Moreover, strain QF2 was isolated from the same soil sample as BartholomewSD, providing support for these findings. Strain QF2 also encoded seven proteins of a Type IE CRISPR-Cas system [67–71]. The QF2 proteins were distantly related to the enzymes of the canonical Cas3 system in *E*. *coli* (Fig 8), but the operon in strain QF2 lacked two genes (Cas1 and Cas2) involved in spacer acquisition. This phenomenon, the absence of Cas1 and Cas2, has previously been reported as a common feature of Streptomycetaceae Type IE systems [72]. The presence in the QF2 genome of a Cas3 system and spacers mapping to essential proteins in each of the genomes of our phages suggests that the strain is likely resistant to all four of our phages, and thus, transduction is unlikely with strain QF2.

**Table 9:** General characteristics of predicted CRISPR-Cas systems within the genomes of strains DF, SFW, QF2, and JS.

Strains	CRISPR	Spacers	Repeats	Spacers with Blastn Hits to Host Range Phage	Cas Loci	Cas-Associated Genes
**DF**	11	13	24	2	3	9
**SFW**	11	23	34	2	3	17
**QF2**	38	161	199	14	5	22
**JS**	4	8	12	0	4	20

Included in this table is the number of spacers within the genome of each bacterial strain with sequence similarity to regions within any of the four phage isolates (IceWarrior, TrvxScott, BartholomewSD, or Shawty).

**Table 10:** Characteristics of the 38 CRISPRs predicted in the draft genome sequence of strain QF2.

Strain QF2						
CRISPRs	Min	Max	Length (nt.)	No. Repeats	No. Spacers	Spacer Blastn Hit to Host Range Phage
1	384313	384428	116	2	1	S1 [BartholomewSD]
2	437656	437755	100	2	1	
3	835820	835922	103	2	1	
4	1307659	1307752	94	2	1	
5	1344898	1345205	308	6	5	
6	1543053	1543144	92	2	1	
7	1616159	1616542	384	5	4	S13 [BartholomewSD, TrvxScott]
8	1618208	1618291	84	2	1	
9	1833873	1833977	105	2	1	
10	1861097	1861197	101	2	1	
11	2316101	2316187	87	2	1	
12	2704894	2704990	97	2	1	
13	3015981	3016376	396	7	6	S21 [IceWarrior]
14	3106815	3107262	448	8	7	S27 [BartholomewSD, TrvxScott]
15	3112452	3113433	982	17	16	S34 [IceWarrior], S41 [TrvxScott]
16	3138610	3140161	1,552	26	25	
17	3145439	3145830	392	7	6	
18	3444685	3444779	95	2	1	
19	3507440	3507598	159	3	2	
20	3791739	3792012	274	5	4	
21	3838730	3838804	75	2	1	
22	4257871	4258080	210	4	3	S89 [IceWarrior]
23	4327550	4328916	1,367	23	22	S105 [IceWarrior], S106 [Shawty, TrvxScott]
24	4333365	4334666	1,302	22	21	S118 [TrvxScott], S131 [Shawty, TrvxScott]
25	4335904	4336481	578	10	9	S138 [BartholomewSD], S140 [TrvxScott]
26	4522773	4522879	107	2	1	
27	4528080	4528148	69	2	1	
28	4657265	4657374	110	2	1	
29	4754273	4754356	84	2	1	
30	4787509	4787642	134	3	2	
31	4987714	4987810	97	2	1	
32	5305650	5305745	96	2	1	
33	5400452	5400541	90	2	1	S151 [IceWarrior]
34	5417083	5417162	80	2	1	
35	5441923	5442032	110	2	1	
36	6552625	6552699	75	2	1	
37	6798173	6798309	137	3	2	
38	7177566	7177797	232	6	5	

Spacers with sequence similarity to any of the four phages in this study are listed next to their corresponding CRISPR and are identified according to their position relative to all other spacers within the QF2 genome.

**Table 11:** Spacers within the genomes of strain DF, SFW, and QF2 that have sequence similarity to at least one of the four phage isolates.

**Strain DF**	** **	** **							
**CRISPR**	**Spacer**	**Blastn Hit to Host Range Phage**	**Score (bits) / E Val.**	**No. Identities (%ID)**	**Strand**	**Minimum**	**Maximum**	**Length (nt.)**	**Sequence**
8	S10	BartholomewSD	30.2 (15) / 2.9	15/15 (100%)	Plus / Minus	4175499	4175530	32	TGC**CCACCGGCCGAGCCG**CCTTCCGCAGGCAG
9	S11	TrvxScott	30.2 (15) / 5.8	15/15 (100%)	Plus / Minus	4689197	4689245	49	GGTGTCCCCGCCGGTCGCGTGCA**TGTCCTTCGGCTTGA**GCGGGCTGCCG
		BartholomewSD	30.2 (14) / 5.8	15/15 (100%)	Plus / Minus				
**Strain SFW**		** **							
**CRISPR**	**Spacer**	**Blastn Hit to Host Range Phage**	**Score (bits) / E Val.**	**No. Identities (%ID)**	**Strand**	**Minimum**	**Maximum**	**Length (nt.)**	**Sequence**
1	S1	Shawty	28.2 (14) / 5.9	14/14 (100%)	Plus / Plus	250625	250650	26	GAGTCACCAGCC**GGGCGAAGGCACGC**
5	S6	IceWarrior	32.2 (16) / 0.99	19/20 (95%)	Plus / Plus	4553831	4553872	42	CGGGCGTCGACGGTGACGAGCG**TCGCGTCGTACTTCTCCTTG**
**Strain QF2**		** **							
**CRISPR**	**Spacer**	**Blastn Hit to Host Range Phage**	**Score (bits) / E Val.**	**No. Identities (%ID)**	**Strand**	**Minimum**	**Maximum**	**Length (nt.)**	**Sequence**
1	S1	BartholomewSD	32.2 (16) / 1.2	16/16 (100%)	Plus / Plus	384347	384394	48	GCGGACGGCG**GCGCGGCCGGTACCCC**CGGTGTCCACGACGGCGGCGCG
7	S13	BartholomewSD	32.2 (16) / 1.9	16/16 (100%)	Plus / Plus	1616356	1616423	68	CGACCTGCGGTACCACTCGATCCGGGCGCGGTCCCATCTA**CAAGGGCACGGTCGTC**CAGCGGACCGAG
		TrvxScott	32.2 (16) / 1.9	16/16 (100%)	Plus / Plus				
13	S21	IceWarrior	30.2 (15) / 2.5	15/15 (100%)	Plus / Minus	3016077	3016109	33	CGCCGGAACCCTCA**AGGAGGAGAACGGCG**CGGG
14	S27	BartholomewSD	30.2 (15) / 3.1	18/19 (94%)	Plus / Minus	3106902	3106938	37	**AGGGCCTGGCCGTGCGGGG**TGCGGGTGGAGTCGTGGT
		TrvxScott	30.2 (15) / 3.1	18/19 (94%)	Plus / Minus				
15	S34	IceWarrior	30.2 (15) / 2.4	15/15 (100%)	Plus / Plus	3112541	3112572	32	ACAGCGACGTCGCC**TACAACTACGCCGCC**TGG
	S41	TrvxScott	28.2 (14) / 9.5	14/14 (100%)	Plus / Plus	3112961	3112992	32	GGTGCTGAACCCGTCG**GCGGCCGTGAACTT**GT
22	S89	IceWarrior	30.2 (15) / 2.5	15/15 (100%)	Plus / Plus	4257958	4257990	33	CCGCGGGCGTCCTT**CGCCGAGGAGACCCT**GCCC
23	S105	IceWarrior	30.2 (15) / 2.5	15/15 (100%)	Plus / Minus	4328429	4328461	33	CATCAGC**GTCTGAAGCAGCACG**CCCATCGCCTT
	S106	**Shawty**	28.2 (14) / 9.5	14/14 (100%)	Plus / Plus	4328491	4328522	32	TG*GATCGA****GCCGGACG*****GGCACA**TCAGCGGCCC
		*TrvxScott*	28.2 (14) / 9.5	14/14 (100%)	Plus / Minus				
24	S118	TrvxScott	28.2 (14) / 9.5	14/14 (100%)	Plus / Plus	4333692	4333723	32	GCCGCGTCCGG**CTACGGCTACGGCT**CCGCCCC
	S131	**Shawty**	28.2 (14) / 9.5	14/14 (100%)	Plus / Plus	4334483	4334514	32	AACG*CCG****TCCATGAGGCG*****CTG**CGTTTGGCGTC
		*TrvxScott*	28.2 (14) / 9.5	14/14 (100%)	Plus / Minus				
25	S138	BartholomewSD	28.2 (14) / 9.5	14/14 (100%)	Plus / Plus	4336177	4336208	32	AACGCGGCAGCGATGG**CCCGTACGAGCGGC**GG
	S140	TrvxScott	28.2 (14) / 9.5	14/14 (100%)	Plus / Minus	4336299	4336330	32	ATCCTCGCCGTCCA**GACCGCCTCGACGC**AGAT
33	S151	IceWarrior	36.2 (18) / 0.063	18/18 (100%)	Plus / Minus	5400476	5400517	42	GTGGTGGCCTCGCCGACCAGTTG**CTCGGACGCCTGGGCGGCC**

The bold portion of each spacer shares high sequence similarity with a region in the genome of the listed host range phage. In cases where a single spacer mapped to two phages, bold and underlined are used to sequences distinguish the two.

Specific spacers mapping to some of our phages were also discovered within the genomes of strains DF and SFW (but not JS). Strain DF contained two spacers that mapped to sequences within the genome of BartholomewSD, and one of these spacers also shared sequence similarity with a region in TrvxScott (Tables 11 and 12). Strain SFW contained two spacers–one that shared sequence similarity with Shawty and another that mapped to a sequence in IceWarrior (Tables 11 and 13). Both strains DF and SFW encoded proteins containing regions with similarity to the RuvC and HNH endonuclease domains of known Cas enzymes. However, given the limited similarity of these putative proteins to known Cas proteins, further study is necessary to determine if they constitute novel Cas systems. If these systems are functional, we predict that strain DF is resistant to infection by TrvxScott and BartholomewSD, and strain SFW, resistant to Shawty and IceWarrior.

**Table 12:** Characteristics of the 11 CRISPRs predicted in the complete genome sequence of strain DF.

Strain DF						
CRISPRs	Min	Max	Length (nt.)	No. Repeats	No. Spacers	Spacer Blastn Hit to Host Range Phage
1	1494904	1495000	97	2	1	
2	1520300	1520423	124	2	1	
3	1998988	1999080	93	2	1	
4	2017315	2017508	194	3	2	
5	2245640	2245744	105	2	1	
6	2447015	2447128	114	2	1	
7	3772076	3772180	105	2	1	
8	4175415	4175556	142	3	2	S10 [BartholomewSD]
9	4689174	4689268	95	2	1	S11 [BartholomewSD, TrvxScott]
10	4806860	4806947	88	2	1	
11	5687469	5687554	86	2	1	

Spacers with sequence similarity to any of the four phages in this study are listed next to their corresponding CRISPR and are identified according to their position relative to all other spacers within the DF genome.

**Table 13:** Characteristics of the 11 CRISPRs predicted in the draft genome sequence of strain SFW.

Strain SFW						
CRISPRs	Min	Max	Length (nt.)	No. Repeats	No. Spacers	Spacer Blastn Hit to Host Range Phage
1	250601	250674	74	2	1	S1 [Shawty]
2	452415	452685	271	3	2	
3	2779718	2779796	79	2	1	
4	2824197	2824275	79	2	1	
5	4553801	4554168	368	5	4	S6 [IceWarrior]
6	4731148	4731651	504	10	9	
7	5166339	5166424	86	2	1	
8	5317268	5317371	104	2	1	
9	5722922	5723016	95	2	1	
10	6532966	6533033	68	2	1	
11	7183989	7184080	92	2	1	

Spacers with sequence similarity to any of the four phages in this study are listed next to their corresponding CRISPR and are identified according to their position relative to all other spacers within the SFW genome.

A curious feature emerged from our analysis of the CRISPRs within the *Streptomyces* strains. Among the 205 predicted spacers encoded by all four bacterial strains, 18 contained sequence similarity (95–100% identity) with at least one of the four phages (Table 9). The lengths of the matching sequences (100% identity) within bacterial spacers ranged from 14 to 18 nucleotides and accounted for approximately half the length of a typical spacer. Additionally, a single spacer occasionally appeared capable of targeting two distantly related phages. These spacers contained sequences mapping to two distinct genes encoded by different viral genomes. For example, spacer 106 in CRIPSR 23 of strain QF2 is 32 nucleotides in length, and it contains 14 bases that share 100% identity with a region in the TrvxScott tape measure gene. These 14 bases overlap (by 8 nucleotides) with another sequence that is 14 base pairs in length and shares 100% identity with a region within the Shawty genome (Table 11). If these spacers functionally serve to resist infection, our analysis suggests that a single spacer may evolve to efficiently target more than one phage, thus providing broad immunity.

### Susceptibility of *Streptomyces* strains to infection by *S*. *platensis* phages

**Fig 9:**
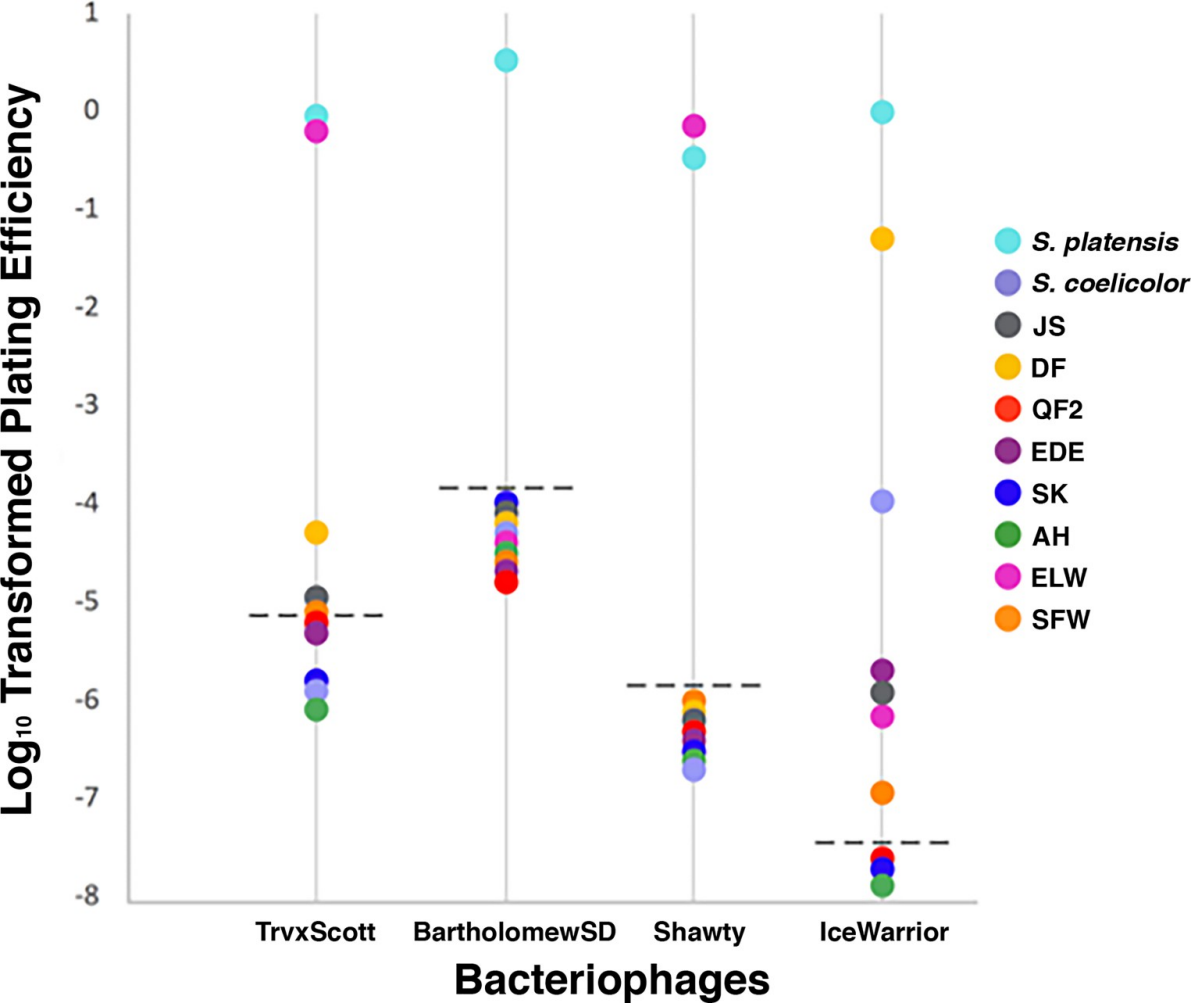
Host ranges of four phage isolates. The phages are listed on the horizontal axis, while the vertical axis indicates plating efficiency (log-transformed). Each circle represents one of ten *Streptomyces* bacteria that was tested for susceptibility to phage infection. Circles above the detection limit (dashed line) represent successfully infected strains of *Streptomyces*.

With the hope of identifying phages that might serve as tools for transduction, we assessed the susceptibility of our *Streptomyces* isolates to infection by each of the four *S*. *platensis* phages (Fig 9). As predicted, strain QF2, with its Type IE Cas system and many CRISPRs containing spacers against our phages, could not be infected by any of our four phages. Strain DF experienced inefficient infection by TrvxScott (~2.0 x 10^4^-fold reduced plating efficiency compared to *S*. *platensis*) and was completely resistant to infection by BartholomewSD. In addition to these results, which were generally predicted by our CRISPR/Cas findings, we also demonstrated strain DF’s resistance to infection by Shawty and susceptibility to IceWarrior (~20-fold reduced efficiency). Strain SFW was at least partially resistant to infection by all four phages: Shawty (no infection), BartholomewSD (no infection), IceWarrior (~10^7^-fold reduced efficiency), and TrvxScott (~1.3 x 10^5^-fold reduced efficiency). Finally, strain JS, despite having no spacers specifically targeting our phages, was similarly immune to infection by Shawty and BartholomewSD and partially resistant to infection by IceWarrior and TrvxScott (~10^6^-fold and ~10^5^-fold reduced efficiency, respectively). These data are consistent with our predictions regarding the resistance of our *Streptomyces* isolates to infection by the phages against which they carry spacers, though it is the case that the presence of a spacer did not always confer complete immunity to the phage it targeted. In some cases, strains containing spacers could be infected relatively inefficiently by the targeted phage. For example, strain DF encoded a single spacer targeting TrvxScott but remained partially susceptible to infection. Strains DF, SFW, and JS were all capable of being infected by TrvxScott and IceWarrior to some degree. Thus, it remains possible that these two phages could be used for transducing BGCs into *S*. *platensis*.

### Identification of phage integrases

In analyzing the proteins encoded within the genomes of our phages, we identified site-specific serine recombinases encoded by BartholomewSD, TrvxScott, and Shawty. The integrases of BartholomewSD and TrvxScott were nearly identical and shared similarity to integrases belonging to a number of previously studied phages. The Shawty integrase shared protein sequence similarity with the integrases of *Streptomyces* phages TG1 and phiC31 (71.3% and 51.9% respectively). The TG1 and phiC31 integrases are distinct recombinases that share 49.7% protein sequence identity and have been used extensively as tools for integrating genes of interest into specific loci within the genomes of a wide variety of organisms, ranging from soil microbes such as *Streptomyces* to multicellular animals such as *Drosophila* [21–25]. Thus, as a newly discovered member of this serine integrase family, the Shawty integrase could also potentially be used to move *Streptomyces*-encoded BGCs between strains to facilitate augmented expression of bioactive natural products.

## Conclusion

This work demonstrates a method for effectively studying newly isolated, antibiotic-producing bacteria and phages that may infect them. We have highlighted how BCP can be used to assess the novelty of BGCs encoded within *Streptomyces* strains, providing another solution to the problem of dereplication. Moreover, our work illustrates how the isolation and genomic analysis of phages that infect antibiotic-producing *Streptomyces* might yield new genetic tools such as transducing phages or integrases, which can be used to augment the expression of novel antibiotics. Specifically, strains DF and JS are promising candidates for future novel antibiotic discovery. Each is active against MRSA and has the potential to produce new chemistries given their encoded biosynthetic arsenal and BCP phenotypes. These two strains are also infected by TrvxScott, which might be used to genetically manipulate their BGCs to induce production of novel antibiotics. Ultimately, our identification of potentially novel BGCs and phage integrases serves as a foundation for further studies that could lead to the discovery of new antibiotics.

## Materials and methods

### Soil sample collection and site description

Undergraduate students enrolled in the Phage Hunters Advancing Genomics and Evolutionary Science (PHAGE) class at UCSD collected soil samples for isolating bacteria and their associated phages. Soil samples (approx. 30 ml) were collected around San Diego County (32.7157° N, 117.1611° W), California, USA. GPS coordinates for the phage isolation samples were: Shawty (32.879232 N, 177.237747 W), IceWarrior (32.963038 N, 117.153242 W), TrvxScott (32.882778 N, 117.243333 W), BartholomewSD (32.881200 N, 117.235000 W). Permits were not required for collection because samples were obtained on the UC San Diego campus and students’ private property/residences.

### Isolation of *Streptomyces*

Actinomycete isolation agar (AIA) plates (for one liter: sodium caseinate 2 g, L-Asparagine 0.1 g, sodium propionate 4 g, dipotassium phosphate 0.5 g, magnesium sulphate 0.1 g, glycerol 5 mL, rifampicin 50 μgmL^-1^, nystatin 100 μgmL^-1^, cycloheximide 100 μgmL^-1^, agarose 15 g, pH 8.1) were used to select for Actinobacteria from soil samples. 1 g of soil was added to the agar surface and streaked across the AIA plate and incubated for two days at 30°C. The plates were investigated for individual colonies with morphologies indicative of *Streptomyces* (vegetative hyphae, aerial mycelium), and those colonies were picked and purified at least four times on AIA plates containing 100 μgmL^-1^ cycloheximide (CHX), which was included to prevent unwanted fungal growth.

### Phage isolation and purification

Actinobacteriophages were isolated from soil samples with host, *Streptomyces platensis* JCM 4664 substrain MJ1A1. An enrichment culture was prepared from 1 g soil and 2.5 ml of *S*. *platensis* added to 15 ml of Luria-Bertani (LB) medium (for one liter: tryptone 10 g, yeast extract 5 g, NaCl 10 g, agar 15 g, pH 7.0), followed by a 2-day incubation at 30°C with shaking. Phage were isolated from a 1.2 ml volume of enrichment culture that was centrifuged at maximum speed for 3 min, 1 ml of the resulting supernatant was filtered (0.22 μM filter), and 5 μl of the filtrate was spotted and then streaked onto an LB plate containing 100 μg/ml of cycloheximide. *S*. *platensis* (0.1 ml) was mixed with 4.5 ml of LB top agar 0.7%, poured over the streak plate and incubated for two days at 30°C. Resultant plaques were re-streaked onto new LB plates containing 100 μg/ml of cycloheximide about 3–4 times for phage purification.

### Bacterial genomic DNA extraction and quantification for 16S rRNA PCR amplification and sequencing

An adaptation of the DNeasy® Blood & Tissue Kit (Qiagen) protocol was used for bacterial genomic DNA extraction. Strains were cultured overnight at 30°C in 5 ml of LB broth while rolling. Cells were pelleted (16,000 x g, 3 min) from 1 ml of culture, re-suspended in 180 μl of lysis buffer (prepared in house), and incubated at 37°C for 45 min after which 25 μl of proteinase K (20 mg/mL) and 200 μl Buffer AL (Qiagen) was added. The samples were vortexed at maximum speed for 20 sec, incubated at 56°C for 30 min, and 200 μl of ethanol (96–100%) was added. The samples were vortexed at maximum speed for 30 sec, added to a DNeasy Mini spin column, centrifuged (16,000 x g, 1 min), and the supernatant was discarded. Buffer AW2 (Qiagen) was added (500 μl), followed by centrifugation (20,000 x g, 3 min). The DNeasy Mini spin column was placed into a sterile 2 ml microcentrifuge tube, and the gDNA was eluted in 100 μl of AE Buffer by centrifugation (20,000 x g, 1 min) following a 1 min incubation at room temperature. The gDNA concentration was quantified (1 μl sample volume) with a Thermo Scientific^TM^ NanoDrop^TM^ One Microvolume UV-Vis Spectrophotometer (840274100) and stored at -20°C.

### Bacterial genomic DNA extraction for PacBio whole-genome sequencing

High molecular weight genomic DNA (20–160 kb) was extracted from four *Streptomyces* strains (DF, SFW, QF2, and JS) with the QIAGEN-Genomic-tip 500/G kit (10262) according to the manufacturer’s protocol for bacteria.

### Bacterial whole-genome sequencing, assembly, and annotation

The genome sequences of four *Streptomyces* strains were generated using the Pacific Biosciences RS II (PacBio RS II) single molecule real-time (SMRT) sequencing platform at the IGM Genomics Center, University of California, San Diego, La Jolla, CA. Linear genome sequences were assembled using the HGAP protocol integrated in the PacBio RS II sequencer (smrt analysis v2.3.0/Patch5) resulting in a variable number (n = 1–95) of contigs per genome, and ranged in size from 5.42 to 7.79 Mb. The mauve contig mover was used to order the contigs of three draft genome sequences (genomes of strains SFW, QF2, and JS) relative to a closely related reference sequence (*S*. *pratensis* ATCC 33331, *S*. *globisporu*s C-1027, and *S*. *parvulus* 2297 respectively). DNA sequencing of strain DF resulted in a single contig and did not require reordering to restore gene synteny, however PacBio sequences were combined with Illumina paired end reads. Illumina sequences were generated from a Nextera genomic library and sequenced using the NextSeq 550 platform with the 300 Mbs kit at the Microbial Genome Sequencing Center (MiGS; Pittsburgh, PA). DNA for Illumina sequencing was prepared using the aforementioned protocol for 16S rRNA sequencing. A hybrid assembly of PacBio and Illumina reads was generated in Geneious Prime (2019.2.3) with the following; (1) Illumina paired end reads were processed using the Geneious Prime workflow ‘best practice for preprocessing NGS reads in Geneious Prime,’ (2) Processed reads were mapped to the PacBio genome using the Geneious assembler with default settings, (3) the resulting consensus sequence was exported (.fasta) for downstream analyses. Gene prediction and annotation were made with the Rapid Annotations using Subsystems Technology (RASTtk) platform [73].

### Phage genomic DNA extraction

5 μl of RNase A and 5 μl of DNase I were added to 10ml of lysate, incubated at 30°C for 30 minutes, and then precipitated overnight at 4°C by the addition of 4 ml of 20% polyethylene glycol 8,000. Samples were centrifuged at 10,000 g’s for 30 minutes, and pellets resuspended in Qiagen PB buffer and DNA isolated using a Qiagen plasmid DNA isolation column as recommended by the manufacturer.

### Phage genome sequencing, assembly, and annotation

Genomic DNA of 4 actinobacteriophages (TrvxScott, BartholomewSD, Shawty, and IceWarrior) was sequenced using the Illumina MiSeq platform at the Pittsburgh Bacteriophage Institute sequencing facility. The genomes were assembled with Newbler and checked for quality with Consed. The whole genome sequences were submitted to GenBank (Acc No. MH669016, MK460245, MK433266, and MK433259). DNA Master was used for annotation, and NCBI BLASTp was used to determine the potential function of gene products. Whole genome sequence comparisons were performed in Phamerator [74].

### 16S rRNA PCR amplification and sequencing

16S ribosomal DNA templates (~1,465 bp) were amplified using Q5 high fidelity PCR (New England Biolabs) with the universal primer set 27F (5’-AGAGTTTGATCCTGGCTCAG-3’) and 1492R (5’-GGTTACCTTGTTACGACTT-3’) [75]. Each PCR mixture (50 μl) contained 100 ng of template gDNA, 500 pmol of each primer, and 200 μM dNTPs. PCR thermocycling conditions were as follows: 30 seconds of initial denaturation at 98°C, 30 cycles of denaturation at 98°C for 10 seconds, annealing for 15 seconds at 60°C, extension at 72°C for 1.5 minutes, and a final extension at 72°C for 5 minutes then held at 4°C. PCR products were purified with the oligonucleotide cleanup protocol as described in the Monarch PCR & DNA Cleanup Kit 5 μg user manual (NEB #T1030). Clean PCR products were sequenced using Sanger methods by Eton Biosciences (https://www.etonbio.com/) and trimmed for quality before analysis.

### CRISPR-Cas sequence analysis and predictions

The sequences of all four *Streptomyces* were searched for CRISPR arrays (repeats and spacers) and potentially associated Cas genes using the following software tools; CRISPR-Cas++ [67, 68], CRISPROne [69] CRISPRDetect [70], and CRISPRMiner2 [71].

### Phylogenetic analyses of bacterial isolates

16S rRNA sequences were trimmed on both ends, (5’ and 3’) in Geneious Prime using the Trim Ends function with an error probability limit set at 0.05, which trims regions with more than a 5% chance of an error per base. Sequences were aligned using MUSCLE v3.8.425 with a maximum of 1,000 iterations, then maximum likelihood was performed using RAxML with 100 rapid bootstrap replicates and the GTR+G model. The tree was visualized using FigTree v1.4.2.

### Cross-streak method for assessing antibacterial production potential

From a single colony, using sterile Q-tips, *Streptomyces* isolates were streaked in a broad vertical line (2 inch) onto LB, and AIA, solid agar plates and incubated for one week at 30°C. The day before the assay, test strains (*E*. *coli* JP313 Δ*tolC*, *B*. *subtilis* PY79, and *E*. *coli* MC4100) were grown in 5 ml of LB and incubated at 30°C overnight while rolling. On the day of the antibacterial screen, the overnight cultures of each test strain were diluted (1:100 in 5 ml LB) and grown to log phase OD_600_ 0.15–0.2 (~1.5 hr at 30°C while rolling). A volume of 10 μl of each test strain was spotted in distinct lines almost to the edge of the *Streptomyces* line at a perpendicular angle. The plates were incubated overnight at 30°C, then investigated for the presence of zones of inhibition which were measured in millimeters.

### Bacterial cytological profiling (BCP) on plates

Fluorescence microscopy and BCP on plates was performed as previously described by Nonejuie et al. [15]. Briefly, *Streptomyces* strains (DF, SFW, QF2, and JS) were streaked in a vertical line down the center of LB, AIA, and ISP2 plates (for one liter: 4.0 g Difco yeast extract, 10.0 g Difco malt extract, 4.0 g dextrose, 20.0 g agar, pH 7.0), incubated for one week at 30°C. The test strain, *E*. *coli* JP313 Δ*tolC*, was prepared and spotted as described above in the cross-streak method. Following a 2 hr incubation at 30°C, a 1.5 x 1.5 cm square (~2.5 cm^2^) piece of agar containing the *E*. *coli* test strain was cut and prepared for high resolution fluorescence microscopy. The cut piece of agar was placed on a microscope slide, the *E*. *coli* cells were stained with fluorescent dyes, a coverslip was placed on top of the stained cells then imaged.

### Host range experiment

The host ranges of 4 phages were determined against the *Streptomyces* strains: QF2, DF, JS, and SFW. The experiment was blinded by assigning phages numbers i-iv and hosts letters A-D. A lawn of *Streptomyces* in LB top agar was poured on LB CHX plates. After the top agar solidified, a grid was drawn on the bottom of the plate, and 5 μl of pre-diluted phage samples (10^0^ to 10^−10^ in phage buffer) were spotted in squares on the grid. Plaques were counted and used to calculate a titer, which was then compared to the titer obtained against *S*. *platensis* to calculate the efficiency of infection.

### Transmission electron microscopy

10 μl of lysate was applied to a copper grid, stained with 1% uranyl acetate, washed twice with phage buffer, and allowed to dry. Images were collected using a FEI Tecnai Spirit G2 BioTWIN Transmission Electron Microscope equipped with a bottom mount Eagle 4k camera.

### Strains used in this study

We used the following strains: *S*. *platensis* JCM 4664 substrain MJ1A1, *E*. *coli* MC4100, *B*. *subtilis* PY79, *P*. *aeruginosa* P4, *S*. *aureus* MRSA USA300 TCH1516 from Texas Children’s Hospital (USA300-HOU-MR), *S*. *coelicolor* A3(2) substrain M146, *E*. *coli* JP313 *ΔtolC*, as well as two strains generously donated by Prof. Keith Poole at Queens University in Kingston, Canada–*P*. *aeruginosa* PA01 and *P*. *aeruginosa* K2733 Δefflux (*ΔMexAB–OprM*, *ΔMexCD–OprJ*, *ΔMexEF–OprN*, *ΔMexXY–OprM*). The Δ*tolC5* mutation is derived from strain EW1b (CGSC #5634), and was introduced into strain JP313 [76] by P1 transduction. JP313 was transduced to tetracycline resistance with a lysate of strain CAG18475 (*metC162*::Tn*10*), and the methionine requirement of the transductants was confirmed. This strain was then transduced to prototrophy with a lysate of EW1b, and these transductants were screened on MacConkey agar for the presence of the Δ*tolC5* mutation. EW1b and CAG18475 were obtained from the *Coli* Genetic Stock Center at Yale University.
