# Supplementary material for: Isolation and characterization of Streptomyces bacteriophages and Streptomyces strains encoding biosynthetic arsenals
Source: PLoS One. 2022 Jan 21;17(1):e0262354. doi: 10.1371/journal.pone.0262354 (PMC8782336; doi:10.1371/journal.pone.0262354)
Supplement: Attachment — Submitted filename: Response to reviewers.docx [file pone.0262354.s001.docx]

Response to reviewers:

Reviewer #2: Major Concerns: My first concern regarding this manuscript is that while the authors deposited the genomic sequence of the isolated Bacteriophages (Actinophages) in GenBank and provided their accession numbers, the genomic sequence of the actual Streptomyces bacterial strains does not seemed to be deposited in the GenBank and I could not find supplemental materials with those sequences (The whole manuscript was in one PDF file). I believe the manuscript should not be published before the authors deposit the Genomic sequences of the Streptomyces strains in GenBank or other similar public database and provide the accession numbers in the manuscript.

Response: We agree and have now submitted the sequence to GenBank. We also verified the sequences before submission by performing Illumina sequencing to ensure the sequences were accurate.

My second concern is that while the manuscript has a large amount of data there is no conclusion provided. I believe the manuscript might benefit from adding a brief conclusion section after the results and the discussion section to present the overall conclusion.

Response: We now have a Conclusion section at the end following the results and discussion.

Minor Concerns: 1. Streptomyces should be Italicized throughout the manuscripts. It was rarely italicized so this needs to be fixed.

Response: We have made this correction.

2. The majority of the tables can be moved to supplementary materials. The manuscript as currently written has so many tables and are almost all just a list of genes so I feel it is more appropriate to move them to the supplementary materials and label them as such. It would be also beneficial to include Excel files of the annotation results if possible.

Response: We agree with the reviewer that there a large number of tables, but since they are the main sources of data we believe they belong in the main body of the manuscript where the information can be quickly accessed by the reader.

3. Bacteriophages would be better referred to as Actinophages in this manuscript since the assumption is that they are phages of Streptomyces which is an actinomycete.

We have made this correction, referring to them as actinobacteriophages. We note some manuscripts refer to them as actinophages and others as actinobacteriophages. We chose to use the term actinobacteriophage since these phage and there genomes are deposited in the actinobacteriophage database run by Graham Hatfull.
