# Supplementary material for: Isolation and characterization of Streptomyces bacteriophages and Streptomyces strains encoding biosynthetic arsenals
Source: PLoS One. 2022 Jan 21;17(1):e0262354. doi: 10.1371/journal.pone.0262354 (PMC8782336; doi:10.1371/journal.pone.0262354)
Supplement: Attachment — Submitted filename: Response to Reviewers.docx [file pone.0262354.s002.docx]

Response to reviewers:

Reviewer #1: In the paper entitled “Isolation and characterization of Streptomyces bacteriophages and Streptomyces strains encoding biosynthetic arsenals”, Pogliano and co-workers describe the isolation of 8 novel strains of Streptomyces, identification of strains that likely produce antibiotics, and proposals of their mechanisms of action using bacterial cytological profiling. Additionally, 4 novel actinobacteriaphage were characterized.

Major concerns:

1. It is generally quite challenging to determine the species of Streptomyces based solely on the 16s rRNA sequencing. Typically, it is better practice to sequence several housekeeping genes (see Antonie van Leeuwenhoek volume 110, pages563–583 (2017) for more details). It would be useful to do this to determine the likely species that these new Streptomyces are.

Response: While we agree about the best practices for generating phylogenetic trees for determining species level taxonomy, here we are only interested in assigning these strains at the genus level.  We sequenced the 16S rRNA genes from the eight soil bacterial isolates to confirm the genus of each was indeed *Streptomyces*. Therefore, we believe the 16S rRNA sequence is sufficient for our purposes.  We did change the legend to more accurately describe our intentions of generating a genus level phylogenetic tree.

2. Why was neighbor joining chosen for the phylogenetic tree? It seems that a maximum likelihood tree might make more sense? This is not a huge dataset so a maximum likelihood tree should be doable.

Response: We agree that a maximum likelihood tree is a better representation of the genus level phylogeny among the bacterial isolates and have generated a new tree using maximum likelihood to replace Figure 1.

Minor concerns:

1. Figure 7 is a little blurry/difficult to read.

Response: We uploaded our figure files to the Preflight Analysis and Conversion Engine (PACE) digital diagnostic tool and have generated a high-quality figure to replace the blurred version.

2. The transition from talking about antibiotics to bacteriophage is a little sudden. Almost feels like they could be 2 different papers.

Response: We have added an additional sentence to clarify the rationale for isolating bacteriophage capable of infecting these *Streptomyces* strains for the purpose of genetically manipulating BGCs for natural product discovery.

Reviewer #2: Montaño et al. manuscript entitled "Isolation and characterization of Streptomyces bacteriophages and Streptomyces strains encoding biosynthetic arsenals", Comments given below:

1. Authors should include the future prospectus or outlooks in the manuscript. In reference to which microbes have better performance.

Response: We have included a section in the conclusions section to include future prospects, specifically noting the microbes of greatest potential for antibiotic discovery.

2. In the manuscript microbes name (as Bionomial nomenclature) should be in italics. Please check Table 1 and in the manuscript.

Response: We have made this correction.

3. In the manuscript lots of tables and figures with same data. So, authors should keep some tables/figs in supplementary.

Response: We agree with the reviewer that there a large number of tables and figures, but since they are the main sources of data, we believe they belong in the main body of the manuscript where the information can be quickly accessed by the reader.  While there is generally no redundancy between the tables, we note that there is a small amount of overlap between Figure 2, which shows the extent of killing of lab strains of *E. coli* and *Bacillus* by strains DF, SFW, QF2 and JS on two different media, and Table 6, which both reports whether or not these strains are able to kill clinical isolates of Gram-negative (*Pseudomonas* PA01 and P4) and Gram-positive pathogens (MRSA).  The data for the lab strains of *E. coli* and *Bacillus* are intentionally included in both tables for reference.
